# Casein Hydrolysate Peptides Attenuate Immunosenescence and Inflammaging in a D-Galactose-Induced Mouse Model of Aging

**DOI:** 10.3390/biology15191741

**Published:** 2026-10-01

**Authors:** Mengni Cui, Zheng Xu, Jianbo Cheng, Jikun Zhang, Caihong Hu, Yun Zhang

**Affiliations:** 1 College of Food Engineering, East University of Heilongjiang, Harbin 150100, China; cuimengni93@outlook.com (M.C.); 18202665997@163.com (Z.X.); hch314159@163.com (C.H.); 2 Key Laboratory of Dairy Science, Ministry of Education, Northeast Agricultural University, Harbin 150036, China; 3 Heilongjiang Feihe Dairy Co., Ltd., Harbin 150100, China; zhangjikun@feihe.com

**Keywords:** casein peptides, aging, oxidative stress, immunological surveillance

## Abstract

Aging populations worldwide face increasing burdens from physiological deterioration. Bioactive peptides derived from milk proteins have attracted interest for their biological activities, yet systematic evidence against aging remains limited. Here, we investigated casein hydrolysates in a D-galactose-induced murine aging model. Following eight weeks of oral administration at graded doses, we quantified systemic oxidative stress markers, inflammatory cytokine profiles, hepatic and cerebral histopathology, and multiple immune competence parameters. Our data demonstrate that casein peptides dose-dependently suppressed lipid peroxidation, elevated endogenous antioxidant enzyme activities, and rebalanced cytokine networks toward an anti-inflammatory state. Concurrently, peptide treatment attenuated tissue lesions in both liver and brain, while restoring thymus and spleen indices, normalizing T-cell subset distributions, and recovering serum immunoglobulin levels. These findings indicate that oral administration of the casein hydrolysate peptide preparation was associated with improvements in redox homeostasis, inflammatory status, tissue integrity, and immune function in D-galactose-induced aging mice. This work provides mechanistic insights into casein peptides as a multi-target protective agent against age-associated physiological decline, supporting further exploration of their therapeutic potential in aging biology.

## Simple Summary

Aging is a growing global concern, and finding natural ways to support healthier aging is increasingly important. In this study, we investigated whether small protein fragments from casein, a major protein in milk, could help counteract signs of aging. We used a mouse model where aging was induced by D-galactose injections and then treated the mice with different doses of casein peptides for eight weeks. Our results showed that the peptides worked in a dose-dependent manner, reducing oxidative stress, lowering inflammation, and protecting liver and brain tissues from damage. They also helped restore a balanced immune system by improving immune organ health and normalizing immune cell and antibody levels. These findings suggest that casein-derived peptides, as a natural food ingredient, have the potential to support multiple aspects of health during aging and could be explored as a dietary strategy for age-related health conditions.

## 1. Introduction

Aging is an inevitable physiological process characterized by progressive decline of tissue and organ functions, closely associated with increased risk of chronic diseases such as neurodegenerative disorders and immune deficiency [1]. The global population aging trend has accelerated, posing severe challenges to healthcare systems. This trend has significant implications for society and the economy, as evidenced by studies that highlight the increased healthcare burden and the need for innovative strategies to promote healthy aging and mitigate the impact on healthcare systems [2]. Nine hallmarks of aging are widely recognized, with oxidative stress and immunosenescence as core interrelated mechanisms—oxidative stress causes biomolecular damage [3], while immunosenescence impairs immune responses [4], and both form a vicious cycle with chronic low-grade inflammation to accelerate aging [5]. The D-galactose-induced aging animal model is widely used due to its simplicity, stability, and similarity to natural aging, as chronic D-galactose administration accumulates advanced glycation end products, induces oxidative stress, activates inflammation, and reproduces aging phenotypes [6]. Several therapeutic and nutritional strategies have been explored to alleviate D-galactose-induced aging-related pathological changes. These approaches mainly include antioxidant supplementation, anti-inflammatory interventions, enhancement of endogenous antioxidant defense systems, modulation of mitochondrial function, and regulation of aging-related signaling pathways such as Nrf2 and NF-κB. In addition, compounds that support cellular energy metabolism, including nicotinamide adenine dinucleotide (NAD^+^)-related interventions, have also been investigated in experimental aging models. Although pharmacological approaches may exert relatively strong and targeted effects, their long-term use for preventive purposes may be limited by safety considerations, cost, and the need for medical supervision. In contrast, health foods and food-derived bioactive components can potentially be incorporated into the daily diet before overt age-related dysfunction develops, providing a comparatively mild and sustained nutritional strategy for maintaining redox balance, controlling low-grade inflammation, and supporting immune homeostasis. Therefore, identifying safe food-derived bioactive substances with multi-target regulatory properties is of particular interest for healthy-aging interventions. Among such food-derived functional components, bioactive peptides from dietary proteins, particularly milk-derived casein, have attracted increasing attention because of their antioxidant, anti-inflammatory, and immunomodulatory activities. These peptides can be released through enzymatic hydrolysis or fermentation and may provide a multi-target nutritional approach to counteract several interconnected features of aging [7]. However, most existing studies focus on single biological activities, lacking systematic investigations on comprehensive anti-aging effects and the crosstalk between oxidative stress, inflammation, and immune function [8]. Based on this, we hypothesized that casein hydrolysate peptides exert anti-aging effects by integrating antioxidant, anti-inflammatory, and immunomodulatory pathways, and this study aimed to prepare and identify casein peptides, evaluate their effects on aging mice’s general status, oxidative stress, inflammation, tissue damage, and immune function, providing experimental evidence for developing casein peptide-based anti-aging products [9].

On the other hand, casein peptides, as functional food ingredients with favorable safety profiles, have been confirmed by multiple animal studies to be safely applicable for in vivo intervention. It has been reported that casein-derived bioactive peptides, owing to their natural origin, good biocompatibility, and low toxicity, can be safely utilized in functional foods and nutraceutical products. Previous studies have shown that casein hydrolysates and casein-derived bioactive peptides possess antioxidant and anti-inflammatory properties and can exert free-radical-scavenging and cellular antioxidant effects [10]. In addition, available toxicological evidence suggests that selected casein hydrolysates can be well tolerated in experimental animals at relatively high oral doses [11]. These findings provide a basis for investigating casein-derived peptides as food-derived bioactive components in vivo. Therefore, in the present study, we employed a D-galactose-induced aging mouse model to systematically evaluate the effects of casein hydrolysate peptides on oxidative stress, inflammatory responses, tissue injury, and immune function.

## 2. Materials and Methods

### 2.1. Preparation and Identification of Casein Peptides

#### 2.1.1. Preparation of Casein Hydrolysate Peptides

The membrane-separated casein liquid prepared by ceramic membrane filtration or spiral-wound membrane filtration was used as the sole substrate for enzymatic hydrolysis in this study. The composition (mass fraction) of the substrate was determined as follows: dry matter accounts for 10.0–12.8%, protein accounts for 84.0–86.9% on a dry basis, casein accounts for 82.0–84.5% of the total protein, and fat accounts for 1.0–5.0% on a dry basis. Four commercial protease preparations were selected for the hydrolysis reaction: Enzyme A is a serine protease derived from *Bacillus licheniformis* with enzyme activity of 300–350 KPROT/g, and the addition amount is 0.001215–0.001836% (mass fraction of casein liquid); Enzyme B is Flavourzyme derived from *Aspergillus oryzae* with enzyme activity of 500–540 LAPU/g, and the addition amount is 0.02013–0.02485% (mass fraction); Enzyme C is Flavourzyme derived from *Aspergillus niger* with enzyme activity of 450–500 U/g, and the addition amount is 0.001096–0.001164% (mass fraction); Enzyme D is Flavourzyme derived from *Aspergillus oryzae* with enzyme activity of 100,000–102,000 U/g, and the addition amount is 0.001082–0.001156% (mass fraction). All enzymes used in the experiment were purchased from well-known commercial suppliers, including Novozymes A/S (Bagsvaerd, Denmark) and Amano Enzyme Inc. (Nagoya, Japan), and all enzymes have passed strict safety assessments and are approved for safe application in food production.

The membrane-separated casein liquor was first pasteurized at 75–85 °C for 15–30 s to inactivate residual proteases and partially denature casein before enzymatic hydrolysis. After pasteurization, the liquor was cooled to 4–6 °C and transferred to an enzymatic hydrolysis tank for temporary storage. It was then heated to the hydrolysis temperature of 58–62 °C with continuous stirring, and the pH was adjusted to 7.7–7.9 using an 8% sodium hydroxide or potassium hydroxide solution. Hydrolysis was carried out for 43–47 min, during which the agitator maintained uniform mixing. Five minutes before the end of hydrolysis, the pH was readjusted to 7.2–7.4 using the same alkaline solution. Upon completion, the hydrolysate was rapidly heated from 58 to 62 °C to 90–95 °C within ≤6 min to inactivate the enzymes, then cooled to 4–6 °C and transferred to a storage tank for further use. The inactivated hydrolysate was concentrated to a dry matter content of 22–25 g/100 g using either a reverse osmosis system or a falling-film evaporation concentrator. Finally, the concentrated hydrolysate was processed into the final product via freeze-drying or spray-drying systems.

#### 2.1.2. LC-MS/MS Identification of Casein Hydrolysate Peptides

For LC-MS/MS analysis, the casein hydrolysate sample was centrifuged at 12,000 rpm at 4 °C for 10 min. The precipitate was discarded, and the supernatant containing approximately 5–8 mg of protein was collected and mixed with sample buffer. The sample was purified using a C18 solid-phase extraction cartridge (Thermo Fisher Scientific, Waltham, MA, USA), and the eluted peptide fraction was dried by vacuum centrifugation. The dried peptides were reconstituted in 0.1% formic acid in water and adjusted to a final concentration of 0.1 μg/μL.

The purified peptide samples were separated using an EASY-nLC 1200 nano-flow liquid chromatography (Thermo Fisher Scientific, Waltham, MA, USA) system equipped with a reversed-phase C18 column (150 μm × 150 mm, 1.9 μm). Separation was performed using 0.1% formic acid in water and acetonitrile with gradient elution. Peptides were subsequently analyzed using an Orbitrap Fusion Lumos high-resolution mass spectrometer (Thermo Fisher Scientific, Waltham, MA, USA) equipped with a positive-mode electrospray ionization source (ESI+), with argon used as the collision gas. The mass spectrometer was operated at a resolution of 60,000 over an *m*/*z* range of 400–1200, and spectra were acquired in profile mode.

The raw mass spectrometry data were analyzed using Proteome Discoverer software (version 2.2; Thermo Fisher Scientific, Waltham, MA, USA). Spectra were searched against a bovine casein sequence database containing αS1-casein (UniProt accession P02662), αS2-casein (P02663), β-casein (P02666), and κ-casein (P02668) from Bos taurus. Peptide sequences and associated modifications were obtained from the database search results. Peptides annotated with phosphorylation modifications were classified as phosphopeptides.

### 2.2. Animal Experiment Design

The mice used in this study were provided by Beijing Vital River Laboratory Animal Technology Co., Ltd. (Beijing, China) (strain: C57BL/6JNifdc; age: 6–8 weeks old; sex: male; number: 60). Young adult male C57BL/6JNifdc mice were selected because the D-galactose model is intended to induce aging-like phenotypes in otherwise young animals within a relatively controlled experimental period, thereby allowing evaluation of intervention effects against D-galactose-induced oxidative, inflammatory, and immune alterations. The housing environment was maintained under an independent air-conditioning fan system with adequate ventilation, at a room temperature of 22–24 °C and relative humidity of 28–50%. The laboratory environment was kept clean and hygienic, and the mice had free access to food and water throughout the experiment [Laboratory Animal Production License: SCXK (Beijing, China) 2021-0006]. This study was reviewed and approved by the Harbin Zhongke Sais Experimental Animal Management and Use Committee (Approval No. HRBSCIEC20250801, approved on 25 July 2025). This committee is a duly constituted institutional animal care and use committee (IACUC) operating in compliance with the national standards of China (GB/T 35892-2018) [12]. All experimental procedures involving animals were carried out in accordance with the ARRIVE guidelines and strictly adhered to the 3R principles (Replacement, Reduction, and Refinement) to minimize animal suffering. After 1 week of adaptive feeding, the mice were randomly divided into 6 groups (*n* = 10 per group, 5 mice/cage):

The blank control group (BC) received no treatment, only being given an equal volume of normal saline by gavage. The aging model group (AM) received subcutaneous injection of D-galactose (150 mg/kg/d) daily. The positive control group (PC) received a subcutaneous injection of D-galactose (150 mg/kg/d) + gavage of Nicotinamide Mononucleotide (NMN) (dose: 200 mg/kg/d, consistent with common anti-aging experimental dosage) daily. The Low/Medium/High-dose casein hydrolysate peptide groups (LD/MD/HD) were given subcutaneous injection of D-galactose (150 mg/kg/d) + gavage of casein hydrolysate peptides (40 mg/mL stock solution) daily, with doses of 100 mg/kg/d (50 μL), 200 mg/kg/d (100 μL), and 400 mg/kg/d (200 μL), respectively (adjusted based on ~20 g body weight of mice).

**Figure 1:**
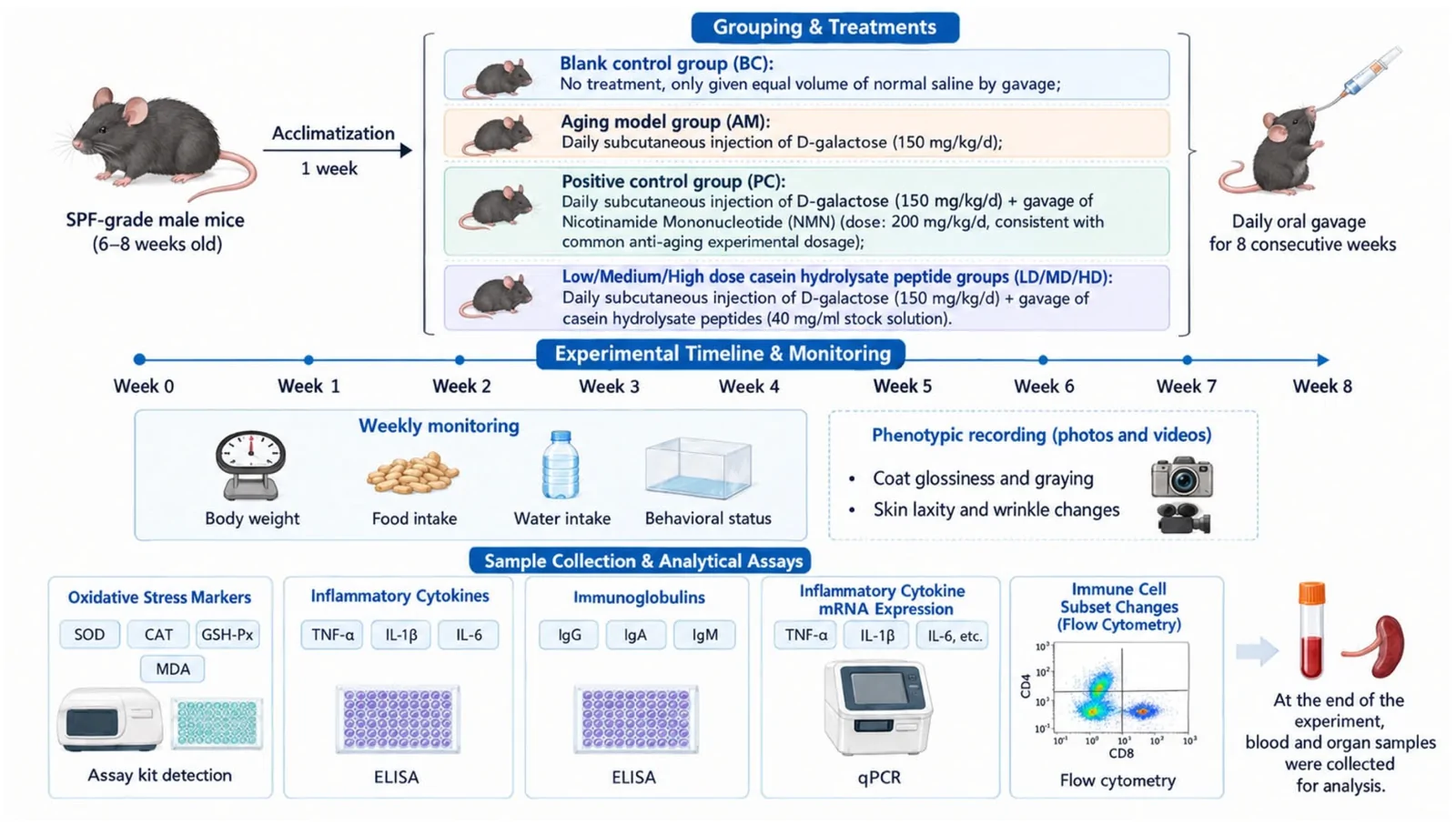
Overall experimental design of this animal study. Stress markers including relative expression levels of superoxide dismutase (SOD), catalase (CAT), glutathione peroxidase (GSH-PX), and malondialdehyde (MDA) in tissues of mice from each group.

The aging model induction and intervention were performed simultaneously, once daily for 8 consecutive weeks. Gavage doses and cycles were determined by pre-experiments to avoid excessive stimulation to mice. The subsequent sampling, detection, and operational procedures performed on the animals are illustrated in Figure 1, which presents the overall experimental design of this animal study.

### 2.3. Sample Collection and Processing

Following the 8-week intervention period, mice were anesthetized with isoflurane for terminal blood and tissue collection. Adequate anesthesia was confirmed by reduced responses to external stimuli, muscle relaxation, and stable reduced respiratory activity, in strict compliance with the regulations on experimental animal welfare and the 3R principles (Replacement, Reduction, Refinement). Before euthanasia, mice were fasted for 12 h with free access to water to minimize potential interference from food on biochemical measurements. Whole blood was collected via orbital venous plexus puncture and placed in sterile centrifuge tubes. The blood samples were allowed to clot at room temperature for 2 h, and then centrifuged at 3000 r/min for 15 min at 4 °C. The upper serum layer was carefully aspirated using sterile pipettes (Guangzhou Jet Bio-Filtration Co., Ltd., Guangzhou, China), aliquoted into sterile EP tubes (100 µL per tube), and stored at –80 °C for subsequent immunological and inflammatory assays.

Under sterile conditions, the abdominal and thoracic cavities were opened, and the liver, spleen, and thymus were rapidly excised, with adherent fascia and blood vessels carefully stripped using sterile forceps (Guangzhou Jet Bio-Filtration Co., Ltd., Guangzhou, China). The cranium was then opened to isolate the whole brain. Each tissue was rinsed with pre-cooled sterile normal saline to remove residual blood, blotted dry with sterile filter paper, and divided into 2–3 aliquots (approximately 50–100 mg each). For the liver and brain, samples designated for histopathological examination were fixed in 4% paraformaldehyde, whereas samples designated for biochemical or gene-expression analyses were rapidly frozen and stored at −80 °C. Spleen and thymus samples were allocated either for frozen storage or for subsequent flow-cytometric analysis according to the experimental design. Specifically, liver samples were allocated for frozen storage (*n* = 7) or fixation (*n* = 3), spleen samples for frozen storage (*n* = 7) or flow cytometry (*n* = 3), brain samples for frozen storage (*n* = 7) or fixation (*n* = 3), and thymus samples for frozen storage (*n* = 7) or flow cytometry (*n* = 3). For selected resource-intensive downstream analyses, three animals were randomly selected from each experimental group for analysis. This subset size reflected the allocation of tissue samples across multiple experimental endpoints and practical constraints associated with these assays.

All sample collection and processing procedures were conducted on an ultra-clean workbench and carried out on ice to prevent sample degradation. Each sample was clearly labeled with the group designation, mouse identification number, and collection date to ensure complete traceability throughout the study.

### 2.4. Assay Indicators and Methods

#### 2.4.1. General Observations, Body Weight, and Food Intake

At a fixed time each day (9:00–10:00 a.m.), the mice were placed in a standard observation cage and video-recorded for 10 min. The recorded videos were subsequently reviewed to assess mental state, activity level, gait coordination, response to stimuli, and social behavior. These observations were used to characterize the general physiological status and aging-related manifestations of the animals.

Body weight was measured individually once weekly using an electronic balance (Shanghai Sunny Hengping Scientific Instrument Co., Ltd., Shanghai, China) (accuracy, 0.01 g) throughout the 8-week experimental period. Food intake was measured at the cage level. At the beginning of each 24 h monitoring period, the amount of food provided to each cage was weighed and recorded as W1. After 24 h, the remaining food was weighed and recorded as W2, and food consumption per cage was calculated as (W1−W2). Because five mice were housed in each cage, the average daily food intake was expressed on a per-mouse basis by normalizing the cage-level food consumption to the number of mice in the cage: average daily food intake (g/mouse/day) = (W1−W2)/5. Body weight and food intake were monitored as general physiological indicators during the experimental period.

#### 2.4.2. Oxidative Stress Biomarkers

To evaluate oxidative stress, the activities of total superoxide dismutase (T-SOD), catalase (CAT), and glutathione peroxidase (GSH-Px), as well as the malondialdehyde (MDA) content, were measured in tissue samples using commercial assay kits according to the manufacturers’ instructions. The kits used were the T-SOD assay kit (Cat. No. A001-1), CAT assay kit (Cat. No. A007-1), GSH-Px assay kit (Cat. No. A005-1), and MDA assay kit (Cat. No. A003-1), all obtained from Nanjing Jiancheng Bioengineering Institute (Nanjing, China).

T-SOD activity was determined using the hydroxylamine method, and absorbance was measured at 550 nm. GSH-Px activity was determined based on the consumption of reduced glutathione, with absorbance measured at 412 nm. CAT activity was determined based on the decomposition of H_2_O_2_, with the remaining H_2_O_2_ reacting with ammonium molybdate and absorbance measured at 405 nm. MDA content was determined using the thiobarbituric acid (TBA) method, with absorbance measured at 532 nm. The measured values were calculated according to the corresponding kit instructions.

#### 2.4.3. Serum Inflammatory Cytokines

Serum concentrations of IL-6, IL-1β, and TNF-α were determined using commercial mouse ELISA kits (IL-6, Cat. No. ml063159A; IL-1β, Cat. No. ml106733A; TNF-α, Cat. No. ml002095A; MLBio, Shanghai, China) according to the manufacturer’s instructions. The detection sensitivities were 15.63 pg/mL for IL-6, 11.75 pg/mL for IL-1β, and 7.81 pg/mL for TNF-α. Absorbance was measured at 450 nm, and concentrations were calculated from four-parameter logistic standard curves.

#### 2.4.4. Histopathological Examination

Histopathological analysis of liver and brain tissues: Fresh tissues were immediately fixed in 4% paraformaldehyde or 10% neutral buffered formalin. After gradient ethanol dehydration, xylene clearing, and paraffin infiltration, the tissues were embedded. Slices with a thickness of 4–5 μm were prepared using a microtome (RM2235, Leica Biosystems Nussloch GmbH, Nussloch, Germany) and mounted on glass slides. Sections were deparaffinized in xylene, rehydrated through graded ethanol, stained with hematoxylin, differentiated in acid alcohol, blued, and subsequently counterstained with eosin. After dehydration and clearing, the sections were mounted and examined microscopically for histopathological alterations.

#### 2.4.5. Immune Organ Indices and Flow Cytometry

After sacrifice, the intact pancreas and spleen were aseptically and rapidly removed, rinsed with pre-cooled physiological saline, and dried with filter paper before being precisely weighed (mg). At the same time, the terminal body weight of the mice was recorded (g), and the immune organ index was calculated as follows: Organ index (mg/g) = Organ wet weight (mg)/Mouse body weight (g).

During flow cytometry analysis, spleen and thymus tissues were processed separately to prepare single-cell suspensions. For spleen samples, the tissue was placed on a 200-mesh stainless-steel sieve in a 60 mm culture dish and gently dissociated using the plunger (Shanghai Kindly Enterprise Development Group Co., Ltd., Shanghai, China) of a 5 mL syringe. Cells were rinsed through the sieve with 5 mL of 1× PBS and collected into a 15 mL centrifuge tube. After centrifugation at 300× *g* for 5 min, the supernatant was discarded, and the cell pellet was resuspended in 2 mL of 1× red blood cell lysis buffer (MA0207, meilunbio, Dalian, China) and incubated for 2 min. Lysis was terminated by adding 5 mL of 1× PBS, followed by centrifugation at 300× *g* for 5 min. The cells were washed once more with 5 mL of 1× PBS and resuspended in PBS to obtain a single-cell suspension. For thymus samples, tissues were similarly dissociated through a 200-mesh stainless-steel sieve, washed with 5 mL of 1× PBS, centrifuged at 300× *g* for 5 min, and resuspended in PBS; no red blood cell lysis step was required.

For Fc receptor blocking, prepared cell suspensions were incubated with 0.5 μg anti-mouse CD16/CD32 antibody in 100 μL of 1× PBS at 4 °C for 15 min. Cells were then stained with surface antibodies at 4 °C in the dark for 30–60 min. The antibodies used were PE/Cyanine5 anti-mouse CD4 (BioLegend, San Diego, CA, USA, Cat. No. 100513; 0.3 μL per 100 μL), APC anti-mouse CD8a (BioLegend, Cat. No. 100711; 1.25 μL per 100 μL), PE anti-mouse NK-1.1 (BioLegend, Cat. No. 108707; 1.25 μL per 100 μL), and FITC anti-mouse CD49b (BioLegend, Cat. No. 103503; 0.5 μL per 100 μL). After staining, cells were washed with 1 mL of 1× PBS, centrifuged at 300× *g* for 5 min, resuspended in 0.5 mL of 1× PBS, filtered through a 200-mesh sieve, and analyzed using a NovoCyte 3110 flow cytometer (Agilent, Santa Clara, CA, USA).

#### 2.4.6. Serum Immunoglobulin Measurement

Serum IgG and IgM concentrations were determined using commercial mouse ELISA kits (IgG, Cat. No. ml037601A; IgM, Cat. No. ml063597A; MLBio, Shanghai, China) according to the manufacturer’s instructions. The detection sensitivities of the IgG and IgM assays were 0.13 ng/mL and 15.3 pg/mL, respectively.

Briefly, serum samples and standards were added to the appropriate wells of pre-coated microplates and incubated at 37 °C. After the specified washing and antibody-incubation steps, the chromogenic substrates were added, and the reaction was terminated with stop solution. Absorbance was measured at 450 nm. Sample concentrations were calculated from four-parameter logistic standard curves and corrected for the corresponding dilution factors.

#### 2.4.7. Quantitative Real-Time PCR Analysis

For qPCR analysis of inflammatory cytokine mRNA expression, liver and spleen tissues were collected and stored at –80 °C in RNA preservation solution or liquid nitrogen. Total RNA was extracted from liver and spleen tissues using AG RNAex Pro Reagent (AG21102, AG, Changsha, China). Briefly, tissue samples were ground under liquid nitrogen and homogenized in 1 mL of RNAex Pro Reagent. After incubation at room temperature for 5 min, 200 μL chloroform was added, and the samples were mixed and incubated for a further 5 min. Following centrifugation at 13,000 rpm for 15 min at 4 °C, the upper aqueous phase was transferred to a new tube and mixed with an equal volume of isopropanol. After incubation for 10 min at room temperature, samples were centrifuged at 13,000 rpm for 10 min at 4 °C. The RNA pellet was washed with 1 mL of 75% ethanol and dissolved in 20 μL DEPC-treated water. RNA concentration and purity were assessed using a microvolume spectrophotometer (Thermo Fisher Scientific, Waltham, MA, USA).

First-strand cDNA was synthesized using the Uni All-in-One First-Strand cDNA Synthesis SuperMix for qPCR (AU341, TransGen Biotech, Beijing, China) in a total volume of 20 μL containing 4 μL of 5× SuperMix, 1 μL of gDNA Remover, total RNA, and RNase-free water. Reverse transcription was performed at 50 °C for 5 min and 85 °C for 2 min, followed by holding at 4 °C.

Quantitative PCR was performed using a QuantStudio 3 Real-Time PCR System (Thermo Fisher Scientific, Waltham, MA, USA). cDNA was adjusted to 200 ng/μL before amplification. Each reaction contained 10 μL of 2× SYBR Green qPCR Master Mix, 0.4 μL each of forward and reverse primers, and 8.2 μL of DEPC-treated water, with cDNA added as template. The amplification program consisted of an initial denaturation at 94 °C for 30 s, followed by 40 cycles of 94 °C for 5 s and 60 °C for 30 s. Melt-curve analysis was subsequently performed at 95 °C for 15 s, 60 °C for 60 s, and 95 °C for 1 s. β-Actin was used as the reference gene. Table 1 shows detailed information on the primer sequences used in this study.

### 2.5. Statistical Analysis

One-way and two-way analysis of variance (ANOVA) were used for comparisons between groups, relying on the assumptions of parametric tests such as normality of data, homogeneity of variances, and independence of observations. Upon identifying statistically significant differences in the ANOVA results (*p* < 0.05), subsequent post-hoc tests were conducted to further analyze pairwise comparisons. For comparisons between two groups, an independent samples *t*-test was directly applied. Appropriate post-hoc test methods were selected based on specific experimental design requirements: Tukey’s test was used when sample sizes were equal across all groups, and only all possible pairwise comparisons between groups were needed. When each experimental group required comparison only with a specified control group (e.g., multiple dose groups vs. blank control group), Dunnett’s test was employed. For unequal sample sizes between groups or unplanned pairwise comparisons, the Bonferroni correction method was adopted. For data that did not conform to the assumptions of normal distribution or homogeneity of variance, non-parametric tests were used, such as the Kruskal–Wallis H test. If overall differences existed, pairwise comparisons were performed using the Mann–Whitney U test, with *p*-values adjusted via the Bonferroni method. Graphical presentation of data and final labeling of statistical significance were completed using GraphPad Prism (Version 9.0). All measurement data were expressed as mean ± standard deviation. In the figures, statistical significance was marked with asterisks and hash symbols. Asterisks indicate comparisons between the AM and BC groups (* *p* < 0.05, ** *p* < 0.01, *** *p* < 0.001, and **** *p* < 0.0001), whereas hash symbols indicate comparisons of the PC, LD, MD, and HD groups with the AM group (# *p* < 0.05, ## *p* < 0.01, ### *p* < 0.001, and #### *p* < 0.0001). For analyses based on three biological replicates per group, effect estimates were additionally reported as between-group mean differences with 95% confidence intervals. A sensitivity analysis was performed to characterize the statistical resolution of the small-sample comparisons. For a two-sided independent-group comparison with *n* = 3 animals per group, α = 0.05, and 80% statistical power, the minimum detectable standardized effect size was approximately Cohen’s d = 3.07 (Table S1). Thus, these analyses were primarily capable of detecting very large between-group effects, whereas small or moderate effects may not have been reliably detected.

## 3. Results

### 3.1. Identification of Casein Peptides

Table 2 quantifies peptide composition and functional distribution in the sample. The results indicated that a total of 1796 peptides were identified; 250 were phosphopeptides (accounting for 13.9% of total peptides), indicating that post-translational phosphorylation modification is a common modification in the sample. Phosphopeptides constitute 11.4% of the sample’s total relative intensity, aligning with their 13.9% proportion by quantity, as highlighted in recent proteomic studies. Other functional peptides (non-phosphorylated functional peptides) account for 23.6% of the relative intensity—far higher than their quantity proportion (4.0% functional peptides), implying these non-phosphorylated functional peptides have higher relative intensity in the sample. Although several functional peptides showed relatively high MS signal intensities, these values should be interpreted as semi-quantitative indicators of peptide detection rather than direct measurements of their absolute abundance or contribution to the biological effects observed in vivo.

Table 3 presents a detailed overview of the peptides derived from the casein hydrolysate sample, including their relative intensity, molecular weight, and predicted biological functions. This analysis was conducted using advanced mass spectrometry techniques, which allow for the precise determination of peptide characteristics and facilitate the prediction of their biological roles. Among the identified peptides, YQEPVLGPVRGPPFIIV exhibited the highest relative intensity (10.17%, 1880 Da) and is predicted to possess a range of therapeutic effects, including ACE inhibition, anticancer properties, antimicrobial activity, antithrombotic potential, and immunomodulatory effects, as supported by research on peptide bioactivities.

Several identified peptides corresponded to sequences or sequence regions previously associated with biological activities. For example, TTMPLW, derived from αs1-casein, has been reported to exhibit ACE-inhibitory activity [13], whereas β-casein-derived sequences containing the YQEPVLGPVR and GPVRGPFPIIV motifs have also been associated with ACE inhibition [14]. In addition, casein phosphopeptides have been reported to exhibit antioxidant properties through mechanisms including transition-metal chelation and free-radical scavenging [15]. These previously reported activities provide a biological context for the peptide profile identified in the present casein hydrolysate.

However, the functional annotations of the identified peptides should not be interpreted as direct evidence that the individual peptides exert anti-aging effects in vivo. Rather, because oxidative stress, inflammation, and immune dysregulation are closely associated with aging-related phenotypes, peptides with reported antioxidant, anti-inflammatory, or immunomodulatory properties may plausibly contribute to the overall biological effects of the hydrolysate. This interpretation remains a hypothesis, as the activity, gastrointestinal stability, bioavailability, and in vivo contribution of individual peptide sequences were not directly evaluated in the present study. Therefore, the identified peptides are more appropriately considered candidate bioactive components of the casein hydrolysate rather than confirmed anti-aging effectors. These findings suggest that the hydrolysate contains candidate bioactive peptides that may contribute to its observed biological effects; however, the contribution of individual peptides remains to be established.

### 3.2. Impact of Casein Peptides on the General Health Status of Aging Mice

**Figure 2:**
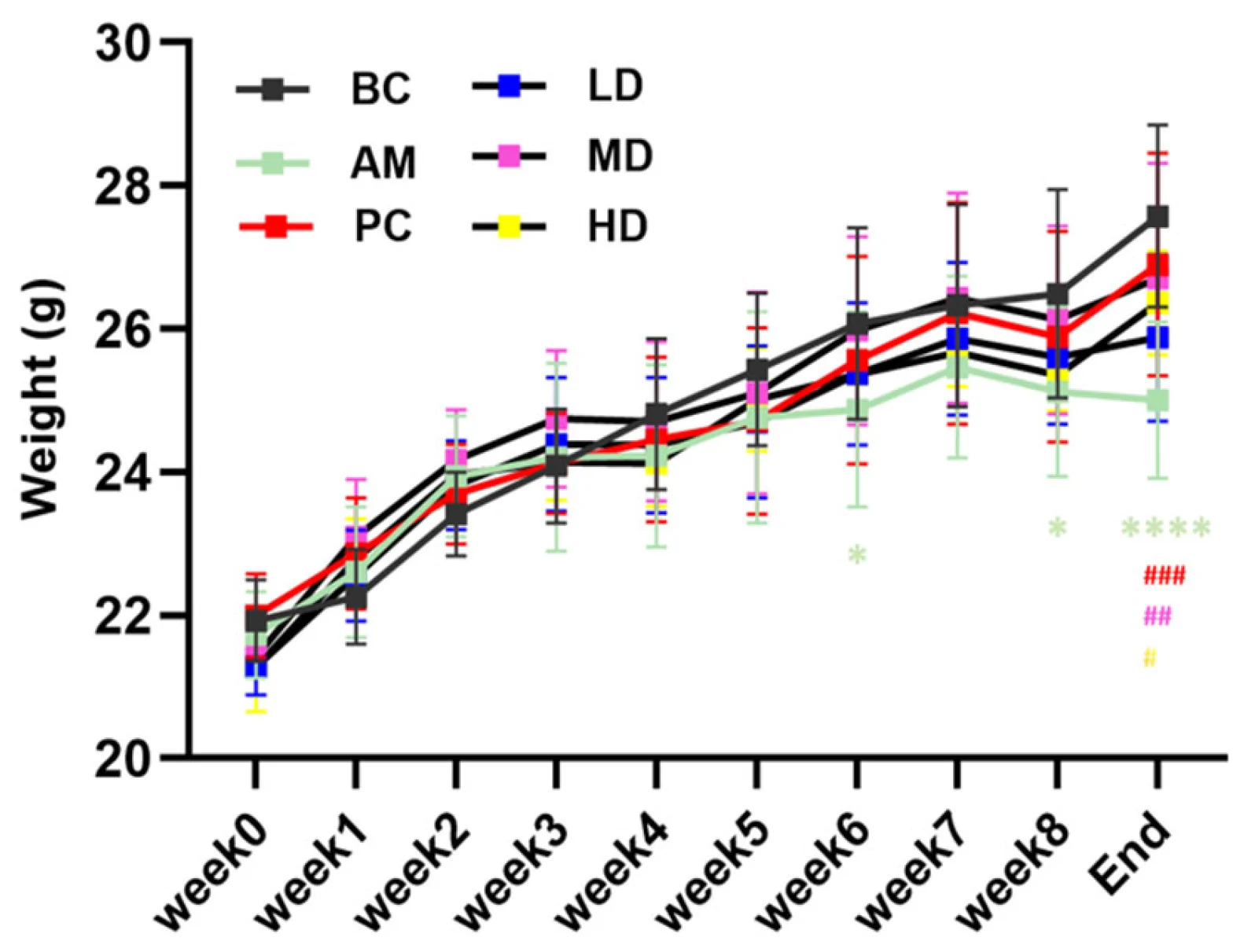
Impact of various dietary interventions on body weight alterations in a mouse model of D-galactose-induced aging. Body weight of mice in each group (BC: blank control; LD: low-dose casein hydrolysate; AM: model control; MD: medium-dose casein hydrolysate; PC: positive control; HD: high-dose casein hydrolysate) was monitored weekly during the 8-week intervention. Body weight was compared among the six experimental groups at each individual time point using one-way ANOVA followed by Tukey’s multiple-comparisons test. Statistical comparisons were performed between groups within the same time point. Data are presented as mean ± SD (*n* = 10 per group). Asterisks indicate comparisons between the AM and BC groups (* p < 0.05 and **** *p* < 0.0001), whereas hash symbols indicate comparisons of the PC, LD, MD, and HD groups with the AM group (# *p* < 0.05, ## *p* < 0.01 and ### *p* < 0.001).

To evaluate the general physiological status of D-galactose-aged mice under dietary interventions, body weight changes were monitored throughout the 8-week experiment (Figure 2). At the initial stage (week 0), there was no significant difference in body weight among all groups. With the progression of D-galactose induction, in the end of the intervention, the body weight of the AM group showed a distinct decreased trend compared to the BC group. Almost no significant differences in body weight were observed among the six groups at the other intermediate time points.

**Figure 3:**
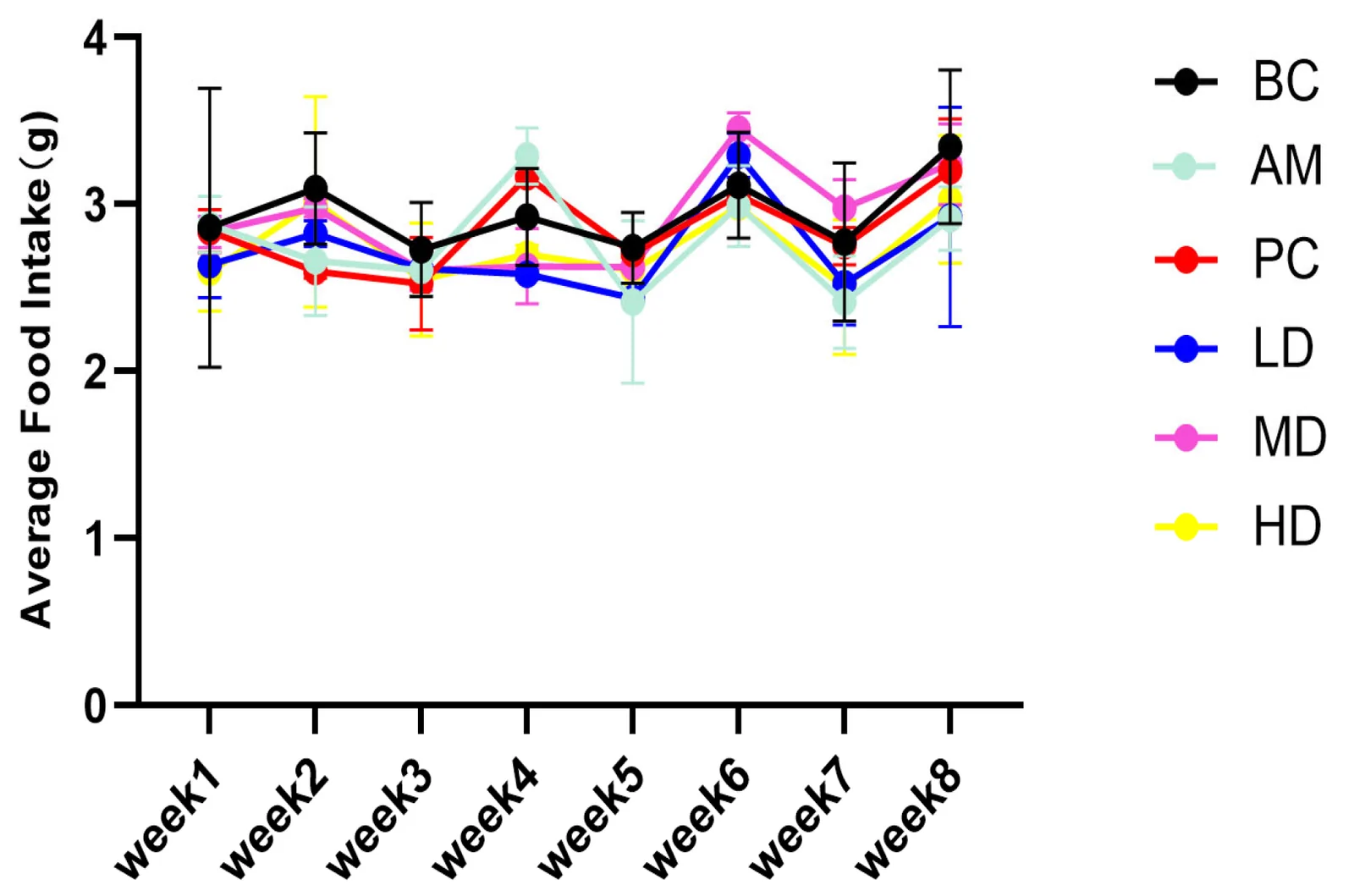
Impact of various interventions on average daily food intake in a mouse model of D-galactose-induced aging. Food intake was measured at the cage level and normalized to the number of mice in each cage. Data are expressed as average daily food intake (g/mouse/day) (BC: blank control; LD: low-dose casein hydrolysate; AM: model control; MD: medium-dose casein hydrolysate; PC: positive control; HD: high-dose casein hydrolysate).

After intervention with casein hydrolysate peptides (LD, MD, and HD groups), at the end time point of Figure 2, the body weight of the intervention groups showed a regulated trend relative to the model group: compared with AM, the body weight of the MD and HD groups exhibited significant differences at this time point, while the LD group showed a similar but less pronounced trend, indicating that casein hydrolysate peptide treatment was associated with differences in body weight compared with the aging model group, particularly at the end of the intervention. Subsequent measurements of food intake and water intake showed no significant differences among the experimental groups. Thus, the observed between-group differences in body weight occurred in the absence of detectable differences in food intake under the conditions of this study (Figure 3).

### 3.3. The Regulatory Effects of Casein Peptides on Oxidative Stress and Inflammatory Markers in Aging Mice

**Figure 4:**
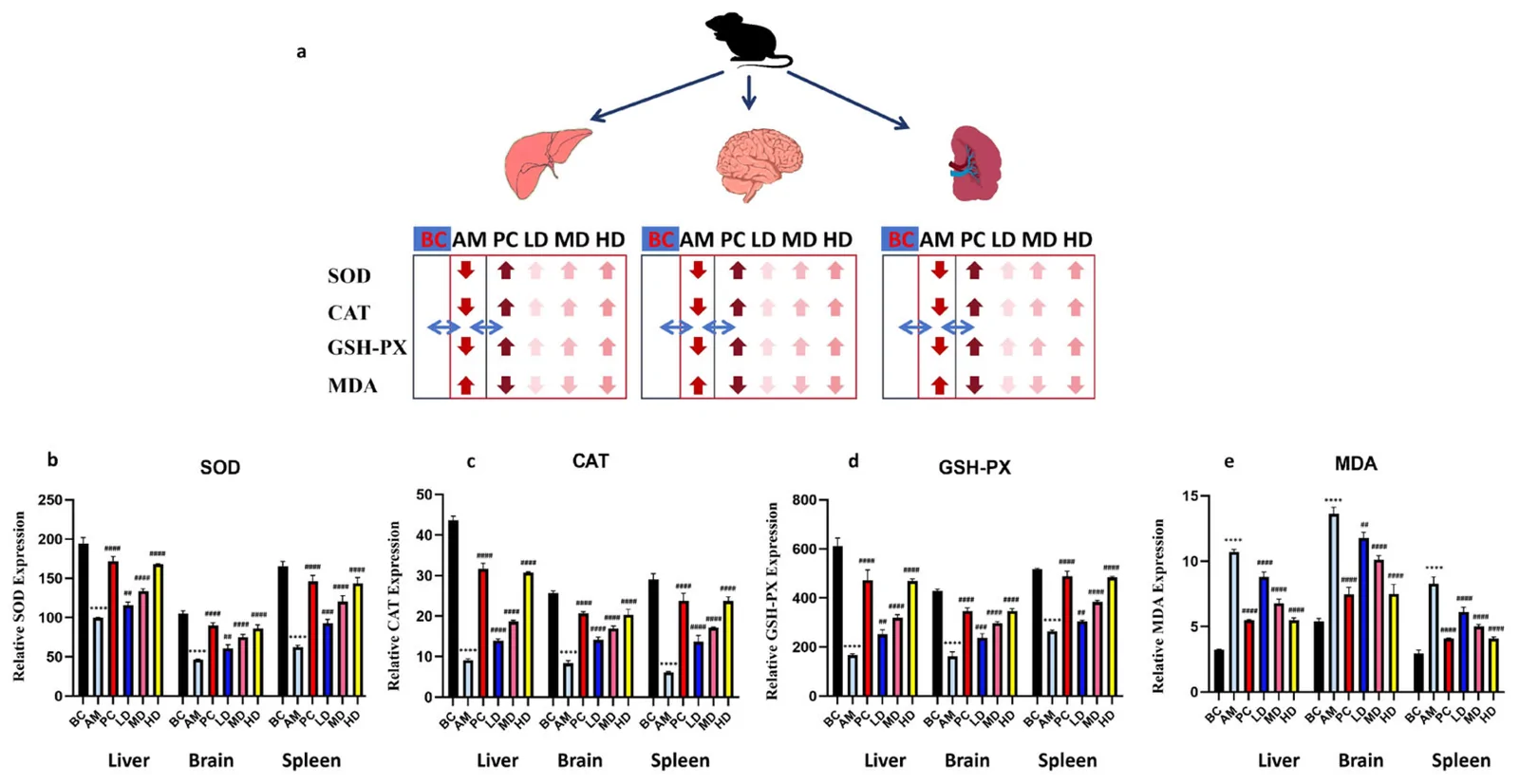
Effects of casein hydrolysate peptides on oxidative stress-related enzyme activities and metabolite levels in D-galactose-induced aging mice. Overview (**a**), SOD (**b**), CAT (**c**), GSH-PX (**d**), and MDA (**e**). The corresponding organs are the liver, the brain, and the spleen, respectively. Relative expression levels of superoxide dismutase (SOD), catalase (CAT), glutathione peroxidase (GSH-PX), and malondialdehyde (MDA) in tissues of mice from each group (BC: blank control; AM: model control; PC: positive control; LD: low-dose casein hydrolysate; MD: medium-dose casein hydrolysate; HD: high-dose casein hydrolysate) after an 8-week intervention. Data are presented as mean ± SD (*n* = 3 per group). Asterisks indicate comparisons between the AM and BC groups (**** *p* < 0.0001), whereas hash symbols indicate comparisons of the PC, LD, MD, and HD groups with the AM group (## *p* < 0.01, ### *p* < 0.001, and #### *p* < 0.0001). Between-group effect estimates as mean differences together with their corresponding 95% confidence intervals have been reported in the Supplementary Table S2.

Hepatic oxidative stress markers were detected to clarify the regulatory effect of casein hydrolysate peptides on D-galactose-induced aging (Figure 4b–e). Compared with the BC group, the AM group showed significantly lower activities of SOD (99.77 ± 0.59 vs. 194.70 ± 6.81 U/mg protein), CAT (9.10 ± 0.32 vs. 43.70 ± 0.94 U/mg protein), and GSH-Px (167.62 ± 5.19 vs. 612.42 ± 31.63 U/mg protein), together with a significant increase in MDA levels (** *p* < 0.0001), indicating marked oxidative imbalance following D-galactose treatment.

Following treatment, the activities of SOD, CAT, and GSH-Px increased in the PC, LD, MD, and HD groups relative to the AM group, whereas MDA levels decreased. For example, hepatic SOD activity increased from 99.77 ± 0.59 U/mg protein in the AM group to 115.80 ± 3.86, 133.63 ± 2.96, 168.08 ± 0.72, and 171.67 ± 6.23 U/mg protein in the LD, MD, HD, and PC groups, respectively. Similar trends were observed for CAT and GSH-Px, with the MD and HD groups generally showing values closer to those of the BC group. MDA levels were also significantly reduced after peptide intervention. Overall, these results indicate a dose-related improvement in hepatic oxidative stress markers following casein hydrolysate peptide treatment. Comparable trends were observed in the spleen and brain. Casein hydrolysate peptide treatment increased antioxidant enzyme activities and reduced MDA levels relative to the AM group, with the medium- and high-dose groups generally showing the greatest changes toward the BC group values. Detailed group comparisons are presented in Figure 4.

### 3.4. Regulatory Effects of Casein Peptides on Inflammatory Factors in Aging Mice

**Figure 5:**
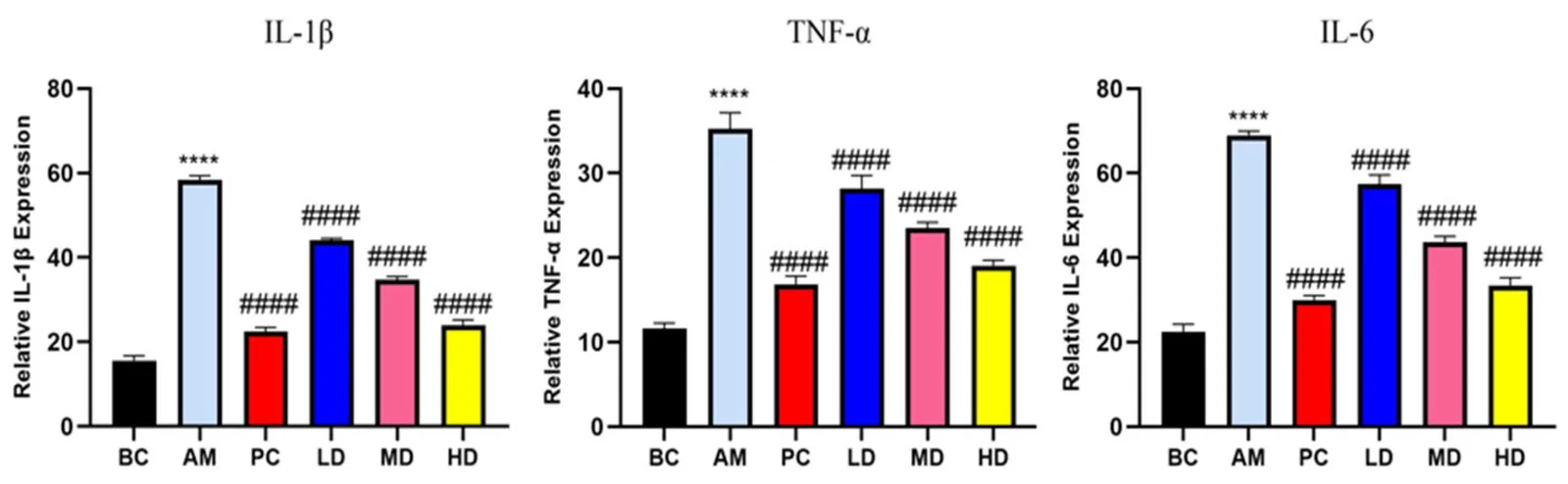
Regulatory Effects of Casein Peptides on Inflammatory Factors in Aging Mice. The relative mRNA expression levels of pro-inflammatory cytokines (IL-1β, TNF-α, IL-6) in tissues of D-galactose-induced aging mice were detected by qRT-PCR. Data are presented as mean ± SD (*n* = 3 per group). Asterisks indicate comparisons between the AM and BC groups (**** *p* < 0.0001), whereas hash symbols indicate comparisons of the PC, LD, MD, and HD groups with the AM group (#### *p* < 0.0001). Between-group effect estimates as mean differences together with their corresponding 95% confidence intervals have been reported in the Supplementary Table S3.

To explore whether casein hydrolysate peptides alleviate aging-related inflammation, we detected the expression of key pro-inflammatory cytokines (IL-1β, TNF-α, IL-6) in aging mice. As shown in Figure 5, compared with the BC group (normal mice), the mRNA levels of IL-1β, TNF-α, and IL-6 in the AM group (D-galactose-induced aging model) were significantly upregulated (**** *p* < 0.0001 (15.55 ± 1.17 vs. 58.41 ± 0.95; 11.60 ± 0.64 vs. 35.24 ± 1.88, 22.40 ± 1.85 vs. 68.98 ± 0.93)), indicating that D-galactose-induced aging was accompanied by a robust inflammatory response.

After intervention with casein hydrolysate peptides (LD/MD/HD groups or PC group), the expression of these pro-inflammatory cytokines was notably downregulated (#### *p* < 0.0001 vs. AM group), and the regulatory effect showed a dose-dependent trend (HD group exhibited the most significant inhibition). Among them, the high-dose group showed the most significant effect (TNF-α: 23.95 ± 1.25; IL-6: 19.06 ± 0.62; IL-1β: 33.42 ± 1.82), which was comparable to that of the positive control group. These results indicate that casein hydrolysate peptide intervention was associated with reduced mRNA expression of the pro-inflammatory cytokines IL-1β, TNF-α, and IL-6 in D-galactose-treated mice, suggesting a potential attenuation of the inflammatory response at the transcriptional level. However, these gene-expression data alone do not establish suppression of specific pro-inflammatory signaling pathways or improvement of immune function.

### 3.5. Protective Effects of Casein Peptides Against Hepatic and Cerebral Histopathological Damage in Aging Mice

**Figure 6:**
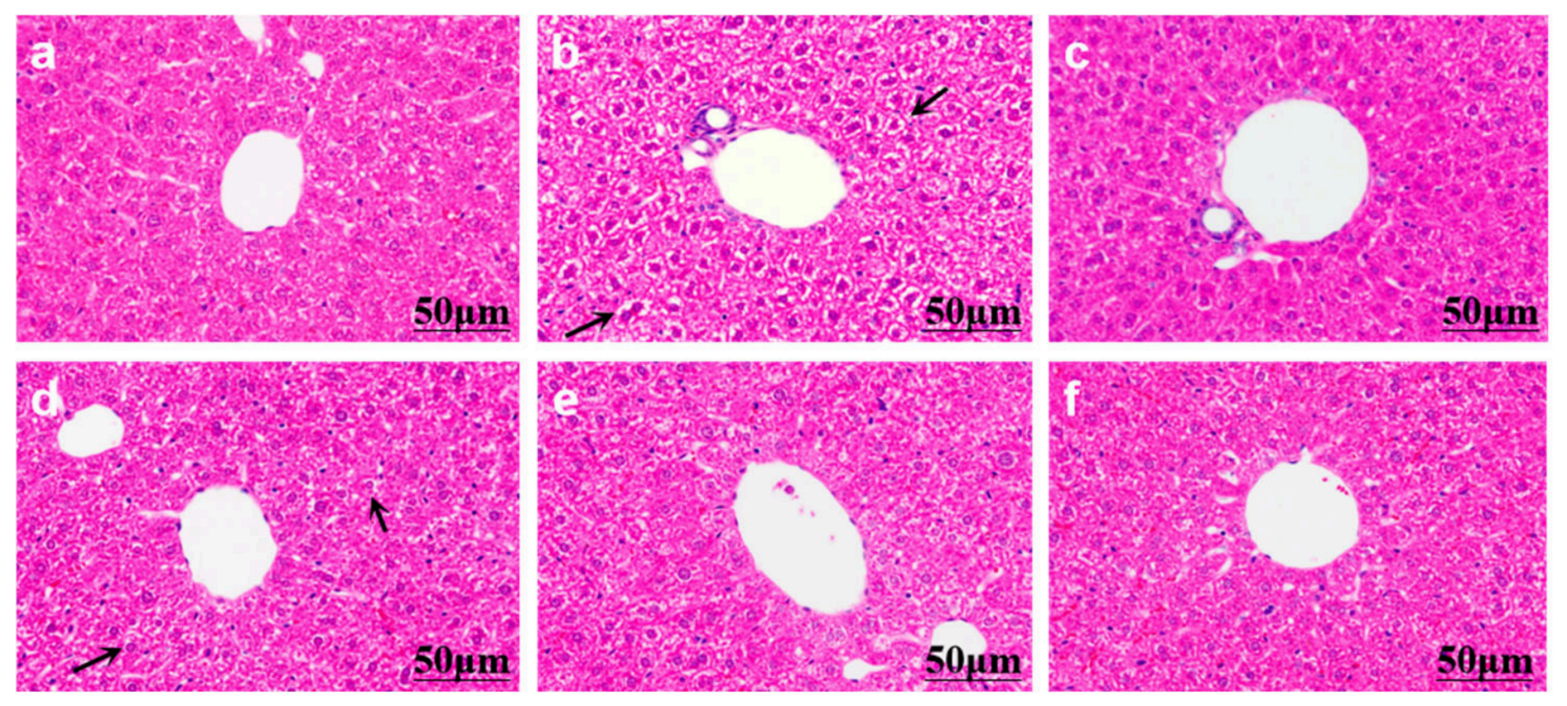
Protective Effects of Casein Peptides Against Hepatic Histopathological Damage (Centrilobular Region) in Aging Mice (H&E Staining, Scale Bar = 50 μm). Representative histopathological sections of liver in different groups: (**a**) BC group (blank control): normal tissue structure with intact cellular morphology (no obvious damage). (**b**) AM group (aging model): severe histopathological damage (arrow: cellular degeneration, inflammatory cell infiltration, structural disorder). (**c**) PC group (positive control): demonstrated enhanced tissue structure and significantly diminished damage compared to the AM group, as evidenced by the protective effects of the substance on liver cells and its ability to reduce liver damage. (**d**–**f**) LD/MD/HD groups (low/medium/high doses of casein hydrolysate peptides, *n* = 3 for each group): gradually alleviated histopathological damage (dose-dependent improvement; HD group shows the most intact structure, close to the BC group).

The centrilobular region (centered on the hepatic central vein) is highly sensitive to aging-related damage. Therefore, its histopathological changes were detected (Figure 6). In the BC group (Figure 6a), the hepatic central vein structure was intact, and surrounding hepatocytes were arranged in a regular radial pattern, consistent with the morphology of normal liver tissue as described in medical literature. In the AM group (Figure 6b), the centrilobular architecture was severely disrupted, with the central vein encircled by disordered, swollen/degenerated hepatocytes and inflammatory cell infiltration, indicative of diffuse, multifocal necrosis and lipidosis, consistent with the hepatic injury induced by D-galactosamine. After intervention, the PC group (Figure 6c) showed partial restoration of the radial hepatocyte arrangement and reduced cellular damage, verifying the positive control’s protective effect.

For the casein hydrolysate peptide groups (Figure 6d–f), the centrilobular damage was alleviated in a dose-dependent manner: the LD group showed mild recovery of hepatocyte organization; the MD group exhibited reduced cellular swelling and infiltration; the HD group presented an intact central vein and radially arranged hepatocytes (structure close to the BC group). These data suggest that casein hydrolysate peptides can specifically protect the hepatic centrilobular region (central vein and surrounding hepatocytes) from D-galactose-induced aging damage, which is a key structural basis for their anti-aging and liver-protective effects.

**Figure 7:**
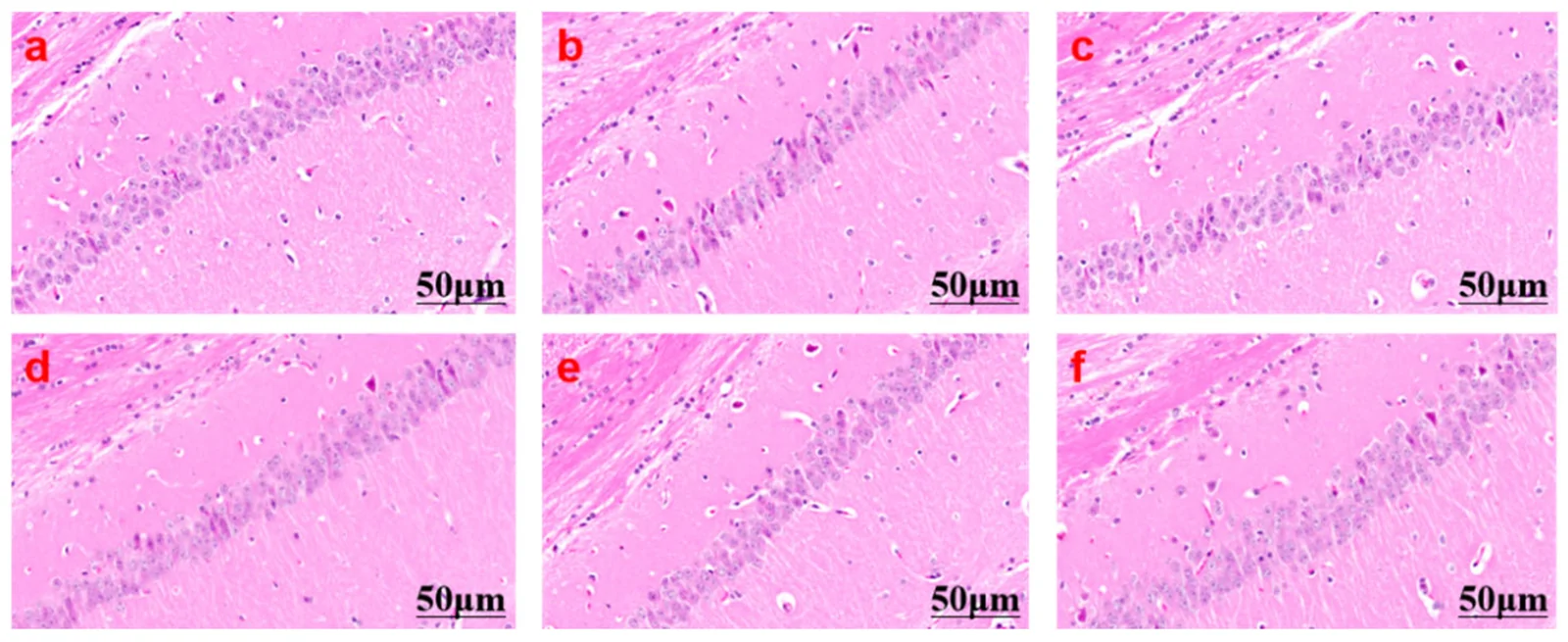
Protective Effects of Casein Peptides on Hippocampal Dentate Gyrus Structure in Aging Mice (H&E Staining, Scale Bar = 50 μm). Representative H&E-stained sections of the hippocampal dentate gyrus granule cell layer in different groups: (**a**) BC group (blank control): Intact granule cell layer with tightly/orderly arranged, plump granule neurons (no degenerative changes). (**b**) AM group (aging model): Markedly thinned granule cell layer (loosely arranged cells; arrow: neurons with nuclear pyknosis); reduced neuronal density and vacuolar changes in the hilus/neuropil. (**c**) PC group (positive control): Improved granule cell layer structure (increased cell density, reduced loose arrangement, fewer pyknotic neurons) compared to the AM group. (**d**–**f**) LD/MD/HD groups (low/medium/high doses of casein hydrolysate peptides, *n* = 3 for each group): Dose-dependent recovery of dentate gyrus structure: LD group shows mildly increased cell density; MD group exhibits reduced neuronal pyknosis; HD group presents tightly arranged granule neurons (close to BC group).

The hippocampal dentate gyrus is critical for neurofunction, so we evaluated its structural changes in aging mice (Figure 7). In the BC group (Figure 7a), the dentate gyrus granule cell layer showed an intact structure: granule neurons were tightly and orderly arranged, with plump morphology and no obvious degenerative changes, consistent with normal hippocampal tissue. In the AM group (Figure 7b), typical aging-related pathological damage was observed: the granule cell layer was markedly thinned with loosely arranged cells, and neuronal density per unit area was significantly reduced (indicating neuronal loss); multiple neurons with nuclear pyknosis were present, and the hilus/adjacent neuropil showed vacuolar changes—reflecting impaired hippocampal microstructure integrity.

After intervention with casein hydrolysate peptides, the above pathological alterations were significantly ameliorated (Figure 7d–f): Compared to the AM group, the granule cell layer exhibited a significant increase in cell density, characterized by a more compact cell arrangement and a reduction in neuronal loss. Despite the presence of a few hyperchromatic cells, the hippocampal structural integrity in the HD group was largely preserved. These results indicate that casein hydrolysate peptides can protect the hippocampal dentate gyrus structure from aging-induced damage, which may contribute to their anti-aging effects via preserving neuronal integrity.

### 3.6. Modulatory Effects of Casein Peptides on Immune Function in Aging Mice

#### 3.6.1. Effects on Immune Organ Indices

**Figure 8:**
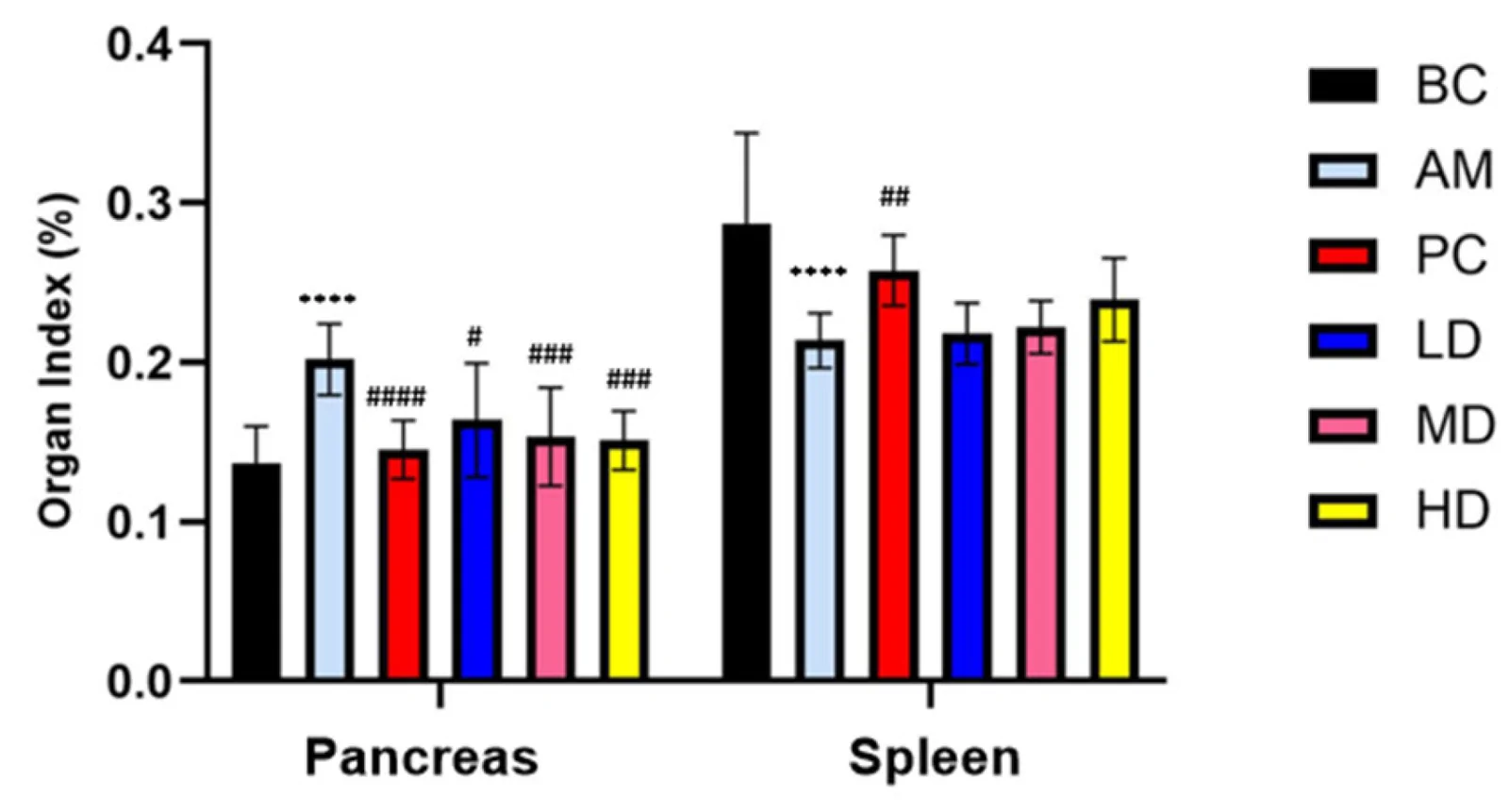
Effects of casein hydrolysate peptides on organ indices. Organ indices of the pancreas and spleen were calculated as organ weight/body weight × 100%. BC, blank control (normal mice); AM, aging model (D-galactose-induced mice); PC, positive control (D-galactose-induced mice treated with the positive-control drug); LD, MD, and HD, D-galactose-induced mice treated with low, medium, and high doses of casein hydrolysate peptides, respectively. Data are presented as mean ± SD (*n* = 10 per group). Asterisks indicate comparisons between the AM and BC groups (**** *p* < 0.0001), whereas hash symbols indicate comparisons of the PC, LD, MD, and HD groups with the AM group (# *p* < 0.05, ## *p* < 0.01, ### *p* < 0.001, and #### *p* < 0.0001).

Organ indices reflect the functional status of immune-related tissues; the pancreas and spleen indices were measured in aging mice (Figure 8). Compared with the BC group, the organ indices of the pancreas and spleen in the experimental group exhibited significant differences, as observed in similar studies with different strains of mice. The values of the AM group (D-galactose-induced aging model) were significantly decreased (**** *p* < 0.0001 for pancreas and spleen), indicating that aging impaired the development/function of these immune-related organs.

After intervention with casein hydrolysate peptides (MD and HD groups), the organ indices of the pancreas were notably restored (### *p* < 0.001 vs. AM group). The recovery effect showed a dose-dependent trend: the HD group exhibited the most significant elevation (close to the BC group level), while the LD group showed mild improvement. These results suggest that casein hydrolysate peptides can reverse the aging-induced reduction of immune organ indices, thereby enhance immune organ function and contributing to the improvement of overall immune capacity in aging mice.

#### 3.6.2. Effects on Immune Cell Subsets

**Figure 9:**
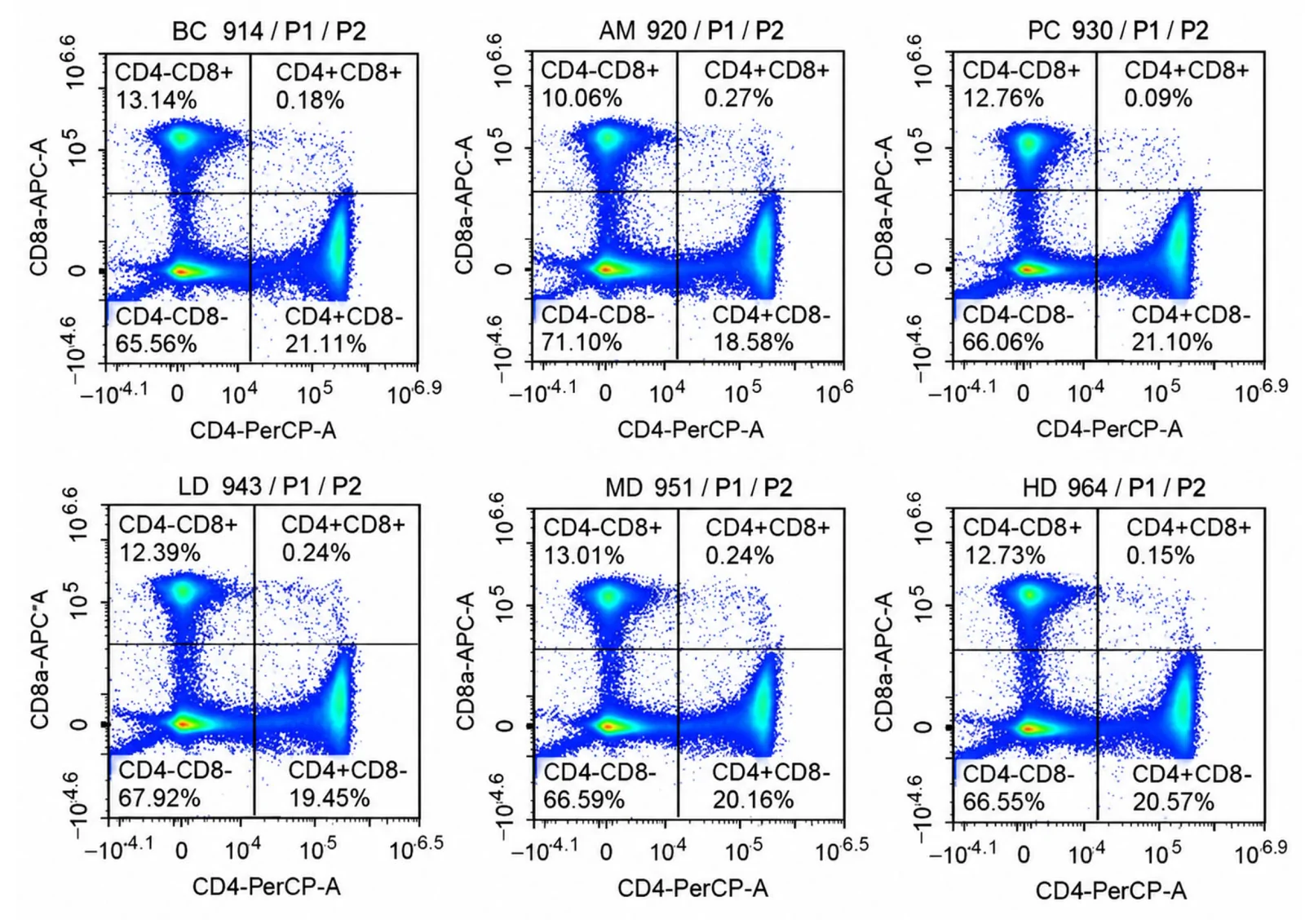
Effects of Casein Hydrolysate Peptides on Splenic Lymphocyte Subsets. Representative flow cytometry plots showing the distribution of splenic lymphocyte subsets (CD4^+^/CD8^+^ T cells) in different groups: BC (blank control), AM (aging model), PC (positive control), LD/MD/HD (low/medium/high doses of casein hydrolysate peptides, *n* = 3 for each group). Plots reflect the proportion of key lymphocyte populations (labeled in plots) in each group.

Figure 9 presents the effect of casein hydrolysate peptides on splenic lymphocyte subsets (a core component of adaptive immunity) via flow cytometry. The balance of lymphocyte subsets is crucial for sustaining immune equilibrium, and disruptions, such as those observed in the AM group, are characteristic of immune dysfunction associated with aging. Studies indicate that casein hydrolysate peptides (LD/MD/HD groups) play a role in modulating immune responses by regulating the distribution of splenic lymphocyte subsets, which can help to counteract the imbalance caused by aging, as evidenced by a shift towards the subset pattern observed in the BC group. This result is important because it directly links casein peptides to the restoration of adaptive immune cell homeostasis, which is a key mechanism underlying their immune-enhancing effects in aging mice.

**Figure 10:**
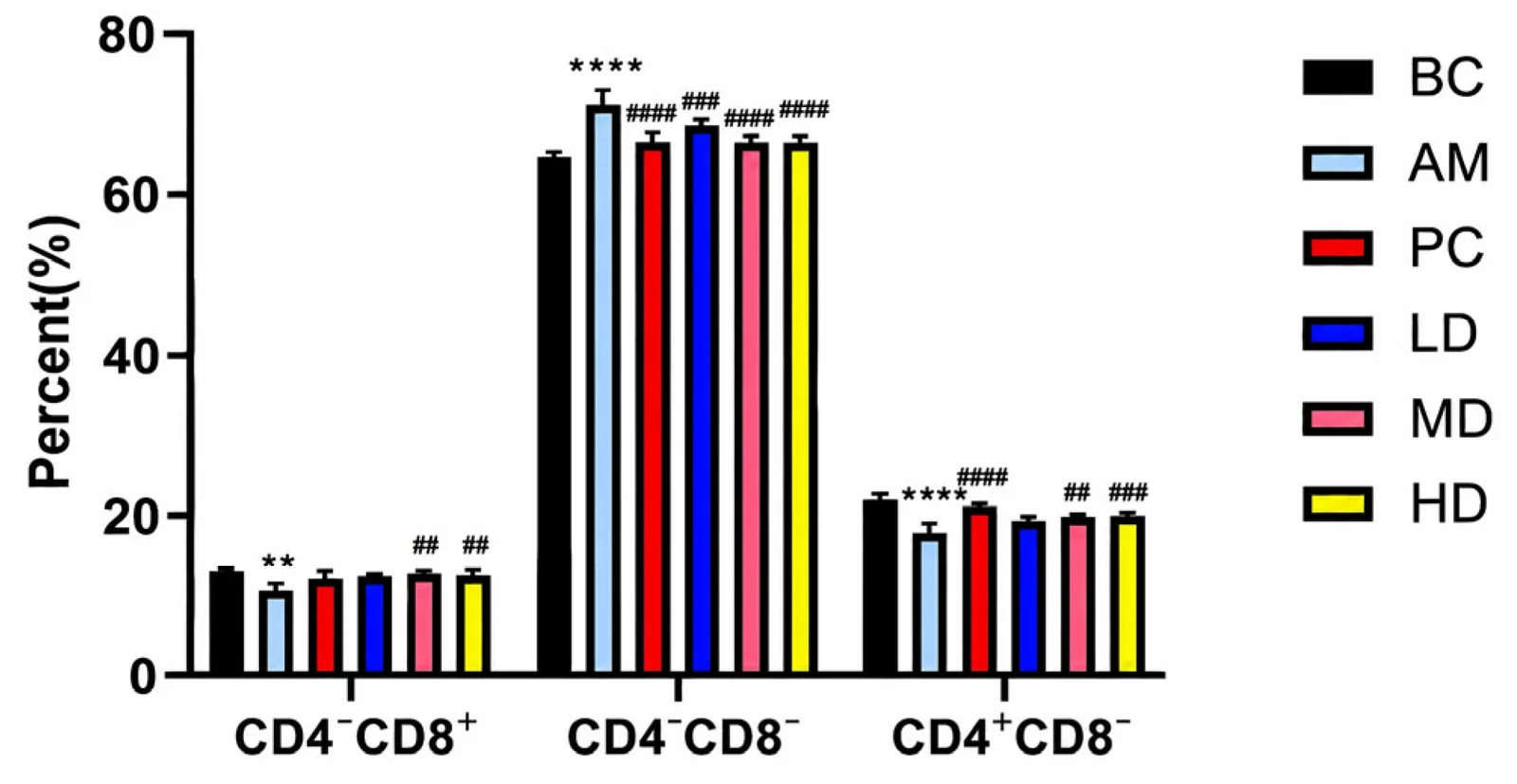
Regulatory Effects of Casein Hydrolysate Peptides on Splenic T Lymphocyte Subset Proportions. Proportions of splenic T lymphocyte subsets (CD4^+^CD8^−^, CD4^−^CD8^+^, CD4^+^CD8^+^) in different groups: BC (blank control, normal mice); AM (aging model, D-galactose-induced mice); PC (positive control, D-galactose-induced mice + positive drug); LD/MD/HD (D-galactose-induced mice + low/medium/high doses of casein hydrolysate peptides). Data are presented as mean ± SD (*n* = 3 per group). Asterisks indicate comparisons between the AM and BC groups (** *p* < 0.01 and **** *p* < 0.0001), whereas hash symbols indicate comparisons of the PC, LD, MD, and HD groups with the AM group (## *p* < 0.01, ### *p* < 0.001, and #### *p* < 0.0001).

T lymphocyte subsets are core mediators of adaptive immunity, and their imbalance is a hallmark of aging-related immune decline. As shown in Figure 10, compared with the BC group (normal immune homeostasis), the AM group showed significant disruption of T lymphocyte subset proportions (e.g., abnormal elevation of CD4^−^CD8^+^ cells, reduced CD4^+^CD8^−^ cells; ** *p* < 0.01, **** *p* < 0.0001 vs. BC), indicating aging-induced immune dysfunction. After intervention with casein hydrolysate peptides (LD/MD/HD groups) or the PC group, the proportions of T lymphocyte subsets were markedly restored to varying degrees, shifting toward the balanced pattern of the BC group. The regulatory effect was dose-dependent (most prominent in the HD group). The proportion of NK cells showed no obvious trend of change, which may be due to sample errors.

These results confirm that casein hydrolysate peptides can correct the aging-induced imbalance of splenic T lymphocyte subsets, thereby restoring adaptive immune homeostasis-this is a key mechanism by which the peptides exert positive effects on the immune function of aging mice.

#### 3.6.3. Effects on Humoral Immunity

**Figure 11:**
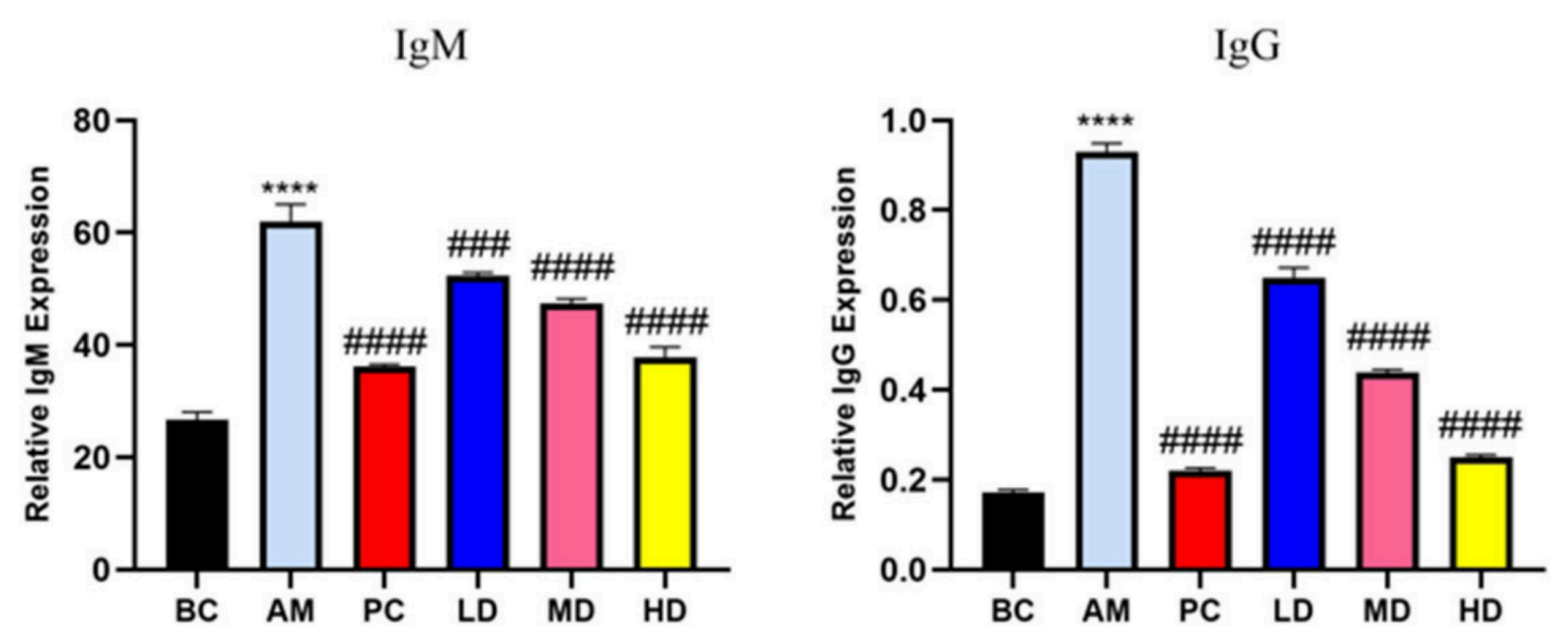
Effects of Casein Hydrolysate Peptides on Serum Immunoglobulin (IgM, IgG) Levels. Relative serum concentrations of IgM (left panel) and IgG (right panel) across experimental groups: BC (blank control, normal mice); AM (aging model, D-galactose-induced mice); PC (positive control, D-galactose-induced mice treated with positive drug); LD/MD/HD (D-galactose-induced mice treated with low/medium/high doses of casein hydrolysate peptides). Data are presented as mean ± SD (*n* = 3 per group). Asterisks indicate comparisons between the AM and BC groups (**** *p* < 0.0001), whereas hash symbols indicate comparisons of the PC, LD, MD, and HD groups with the AM group (### *p* < 0.001 and #### *p* < 0.0001). Between-group effect estimates as mean differences together with their corresponding 95% confidence intervals have been reported in the Supplementary Table S4.

IgM and IgG are core effector molecules of humoral immunity, and their levels directly reflect the functional status of the humoral immune system. As shown in Figure 11, the expression levels of IgM and IgG showed a marked increase in the AM group (61.88 ± 3.21 and 0.92 ± 0.018) compared with the BC group (26.71 ± 1.33 and 0.17 ± 0.005), followed by a gradual decline through the PC, LD, MD, and HD groups.

These findings indicate that D-galactose treatment was associated with altered circulating immunoglobulin levels and that casein hydrolysate peptide intervention partially normalized these changes. However, because antigen-specific responses, immunoglobulin class switching, and memory B-cell responses were not directly assessed, the present data should be interpreted only as changes in serum IgM and IgG levels rather than evidence of isotype switching or humoral immune memory.

#### 3.6.4. Effects on the Expression of Local Inflammatory Cytokine Genes in Immune Organs

**Figure 12:**
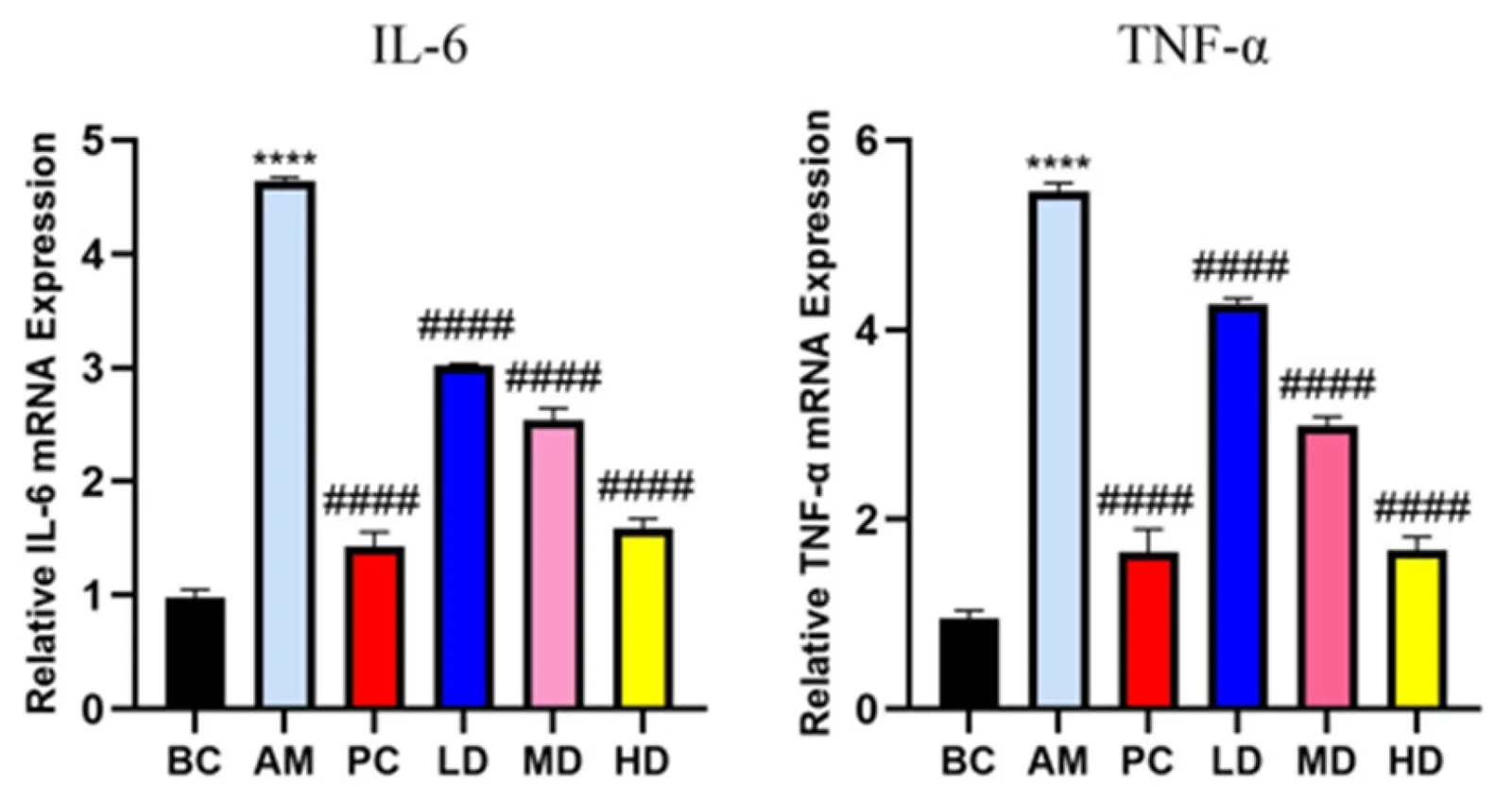
Effects of Casein Hydrolysate Peptides on Pro-Inflammatory Cytokine (IL-6, TNF-α) mRNA Expression in Mouse Liver. Relative mRNA expression levels of IL-6 (left panel) and TNF-α (right panel) in the liver of different groups: BC (blank control, normal mice); AM (aging model, D-galactose-induced mice); PC (positive control, D-galactose-induced mice treated with a positive drug); LD/MD/HD (D-galactose-induced mice treated with low/medium/high doses of casein hydrolysate peptides). Data are presented as mean ± SD (*n* = 3 per group). Asterisks indicate comparisons between the AM and BC groups (**** *p* < 0.0001), whereas hash symbols indicate comparisons of the PC, LD, MD, and HD groups with the AM group (#### *p* < 0.0001). Between-group effect estimates as mean differences together with their corresponding 95% confidence intervals have been reported in the Supplementary Table S5.

IL-6 and TNF-α are key pro-inflammatory cytokines; their excessive hepatic expression drives chronic inflammation (a hallmark of liver aging and damage). Figure 12 shows that compared to the BC group (0.97 ± 0.04 and 0.95 ± 0.05) (low basal inflammatory cytokine levels in healthy liver), the AM group exhibited drastically elevated hepatic IL-6 and TNF-α mRNA expression (**** *p* < 0.0001), indicating D-galactose-induced aging triggers robust liver inflammation. Following the administration of casein hydrolysate peptides (LD, 2.53 ± 0.05 and 4.24 ± 0.03/MD, 3.02 ± 0.008 and 2.98 ± 0.05/HD, 1.43 ± 0.07 and 1.67 ± 0.08 groups) or the PC group, a significant decrease in hepatic IL-6 and TNF-α mRNA levels was observed (#### *p* < 0.0001 compared to the AM group), indicating dose-dependent trend (HD group showed the most significant suppression, approaching BC group levels).

These results suggest that casein hydrolysate peptides have been shown to effectively inhibit the overexpression of hepatic pro-inflammatory cytokines induced by aging, as supported by research on the modulation of cytokine networks. This anti-inflammatory effect in the liver is critical: it alleviates chronic liver inflammation (a driver of hepatic dysfunction in aging) and contributes to systemic anti-aging benefits (since liver-derived cytokines propagate systemic inflammation). Recent studies have shown that peptides, such as chimeric peptide molecules, can effectively target and clear senescent cells, thereby reducing inflammation and supporting the preservation of hepatic homeostasis in aged mice. Additionally, the role of p53 in DNA repair and suppressing inflammation, as mentioned before, may contribute to the peptides’ protective effects against aging-related liver inflammation.

**Figure 13:**
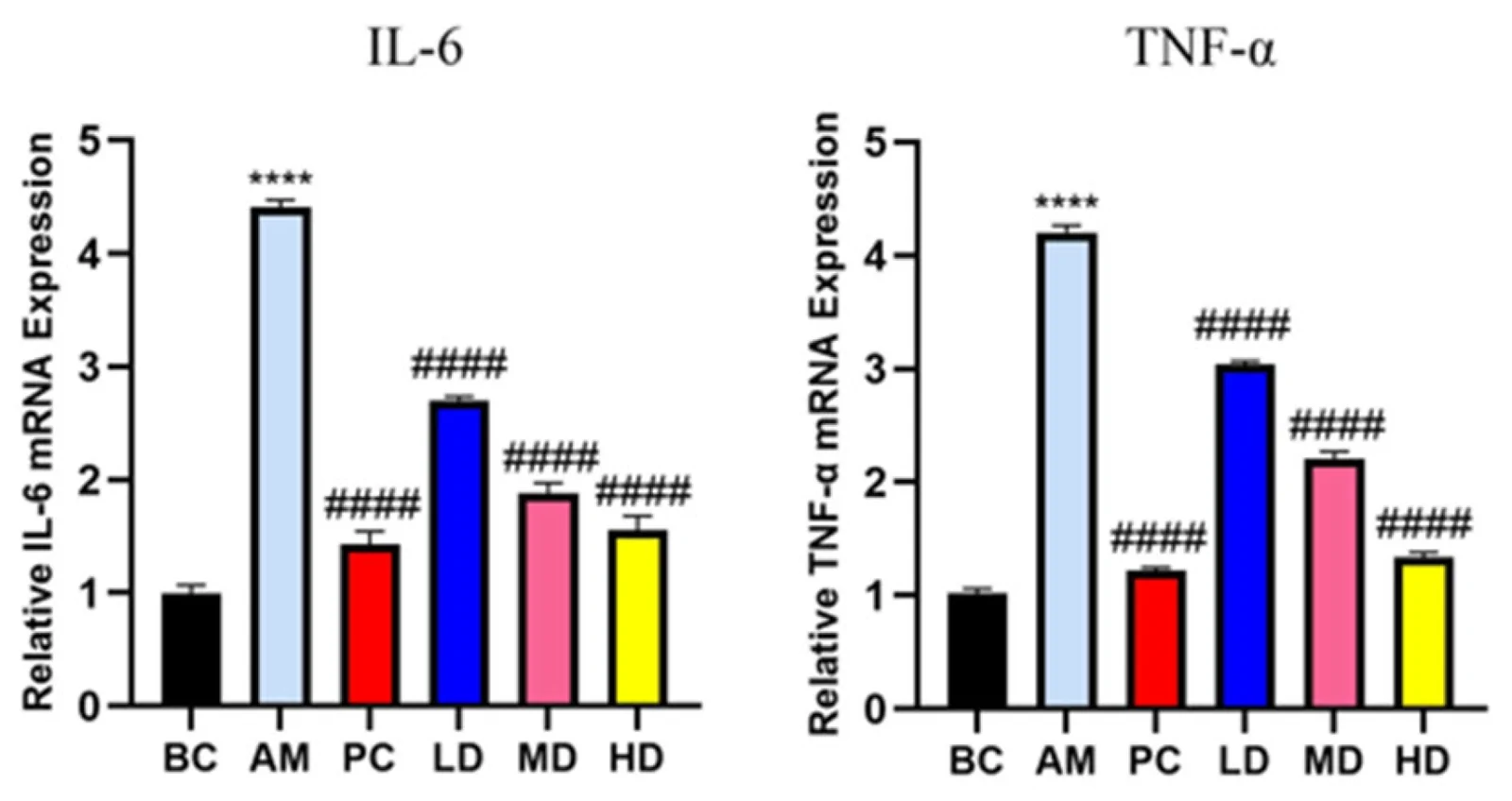
The Influence of Casein Hydrolysate Peptides on the Expression of Pro-Inflammatory Cytokines (IL-6, TNF-α) in Mouse Spleen. Relative mRNA expression levels of IL-6 (left panel) and TNF-α (right panel) in the spleen of different groups: BC (blank control, normal mice); AM (aging model, D-galactose-induced mice); PC (positive control, D-galactose-induced mice treated with a positive drug); LD/MD/HD (D-galactose-induced mice treated with low/medium/high doses of casein hydrolysate peptides). Data are presented as mean ± SD (*n* = 3 per group). Asterisks indicate comparisons between the AM and BC groups (**** *p* < 0.0001), whereas hash symbols indicate comparisons of the PC, LD, MD, and HD groups with the AM group (#### *p* < 0.0001). Between-group effect estimates as mean differences together with their corresponding 95% confidence intervals have been reported in the Supplementary Table S6.

Inflammation, characterized by the production of pro-inflammatory cytokines such as IL-6 and TNF-α, is closely linked to immune dysfunction in the spleen during aging. Figure 13 shows that the AM group (4.41 ± 0.03 and 4.20 ± 0.03) exhibits a significant increase in splenic IL-6/TNF-α mRNA levels compared to the BC group (0.99 ± 0.03 and 1.01 ± 0.02), which aligns with the broader understanding that chronic inflammation, as part of the aging process, can lead to immune system impairment. Casein peptides (LD, 2.70 ± 0.01 and 3.04 ± 0.01/MD, 1.88 ± 0.05 and 2.20 ± 0.03/HD, 1.55 ± 0.07 and 1.33 ± 0.02) and PC reduced these cytokines dose-dependently (HD closest to BC), showing the peptides alleviate splenic inflammation in aging mice, supporting improved immune function. The effect-size analysis showed large between-group differences in hepatic IL-6 and TNF-α mRNA expression, with 95% confidence intervals for the mean differences excluding zero (Table S5 and Table S6). However, these estimates should be interpreted cautiously because only three biological replicates were analyzed per group.

## 4. Discussion

This study systematically evaluated the anti-aging effects of casein hydrolyzed peptides in a D-galactose-induced aging mouse model, confirming that as a multi-target natural active substance, it can improve the overall physiological state of aging mice, alleviate oxidative stress and chronic inflammation, protect important tissues and organs, and enhance immune function, exhibiting potential application value as a functional food ingredient [16]. Through liquid chromatography-tandem mass spectrometry (LC-MS/MS) technology, this study identified a total of 1796 peptides from casein hydrolysate, including 250 phosphoproteins (13.9%) and 72 functional peptides (4.0%). Notably, functional peptides accounted for 35.0% of the total relative intensity, indicating their high relative MS signal intensity in the sample [17]. The representative peptide segment YQEPVLGPVRGPPFIIV (relative intensity 10.17%, molecular weight 1880 Da) was predicted to exhibit various biological activities, such as ACE inhibition and anti-tumor, antibacterial, antithrombotic, and immunomodulatory effects, while phosphoproteins may exert antioxidant effects synergistically through mechanisms such as metal ion chelation and radical scavenging [18]. Although non-phosphorylated functional peptides account for a low proportion in terms of quantity, their high relative MS signal intensity suggests that they may play a dominant role in overall biological activity. This is consistent with computer simulations and in vitro studies reporting high affinity of casein short peptides for aging-related enzymes, supporting the hypothesis that casein hydrolysate exerts a “cocktail” synergistic anti-aging effect [19,20].

In addition, the biological functions listed for the identified peptides were based on reported or predicted activities and should not be interpreted as direct evidence that these individual peptides mediate the anti-aging effects observed in vivo. In particular, several identified peptides have relatively high molecular masses, and their stability during gastrointestinal digestion and their ability to be absorbed intact following oral administration remain unclear. Thus, the identified peptides should be considered candidate bioactive components of the casein hydrolysate rather than confirmed circulating bioactive peptides. Further studies involving simulated gastrointestinal digestion, intestinal absorption and transport assays, and targeted quantitative LC-MS/MS analysis of plasma and tissue samples will be required to determine the stability, bioavailability, and systemic exposure of individual peptides. Accordingly, the biological effects observed in the present study are more appropriately attributed to the casein hydrolysate as a complex peptide mixture rather than to any single identified peptide.

In terms of oxidative stress regulation, D-galactose induction significantly reduced the activities of superoxide dismutase (SOD), catalase (CAT), and glutathione peroxidase (GSH-Px) in the serum, liver, spleen, and brain tissues of aging mice, accompanied by a substantial increase in the level of malondialdehyde (MDA), a product of lipid peroxidation, indicating that the aging model mice were in a state of significant oxidative imbalance [21]. After intervention with casein hydrolyzed peptides, the activities of the aforementioned antioxidant enzymes rebounded in a dose-dependent manner, and MDA content was significantly reduced, with particularly pronounced effects in the medium and high dose groups, consistent with the antioxidant activity trend of casein peptides reported in in vitro studies [22]. The antioxidant mechanism may involve direct scavenging of free radicals, chelation of transition metal ions, and activation of the nuclear factor E2-related factor 2-antioxidant response element (Nrf2-ARE) signaling pathway, which not only drives the transcriptional upregulation of antioxidant enzyme systems but also indirectly participates in the maintenance of telomere length by reducing oxidative DNA damage, thereby delaying the aging process at the molecular level [23].

In addition to the disruption of endogenous antioxidant defenses, the metabolic fate of excess D-galactose should be considered when interpreting the present findings. At high concentrations, D-galactose can be converted by aldose reductase to galactitol, the accumulation of which may contribute to osmotic and redox disturbances. D-galactose, as a reducing sugar, can also participate in non-enzymatic glycation reactions and promote the formation of advanced glycation end-products (AGEs). Engagement of AGEs with RAGE may subsequently activate ROS-sensitive inflammatory pathways, including MAPK and NF-κB signaling, thereby promoting the production of pro-inflammatory cytokines such as TNF-α and IL-6 and contributing to tissue injury. These mechanisms are consistent with the increased oxidative stress, inflammatory cytokine expression, and hepatic and cerebral histopathological alterations observed in the D-galactose model in the present study. Nevertheless, our data do not establish which upstream D-galactose-dependent pathway is primarily responsible for these effects.

An additional possibility raised by the present findings is that interactions between D-galactose and casein-derived peptides may influence the bioavailability or metabolic fate of either component. Because D-galactose is a reducing sugar, glycation or other interactions with amino groups of peptides are chemically plausible; however, whether such interactions occur to a biologically meaningful extent in vivo and whether they reduce, rather than promote, AGE formation remain unknown. The present study did not quantify circulating free D-galactose, intact casein-derived peptides, peptide–D-galactose adducts, AGEs, or RAGE expression. Therefore, the observed reductions in oxidative stress and inflammation cannot currently be attributed to direct sequestration of D-galactose by casein-derived peptides. Future studies using targeted quantitative LC-MS/MS and complementary glycation analyses will be required to determine the in vivo ratios of free D-galactose, bioavailable peptides, and peptide–D-galactose reaction products, together with AGE/RAGE pathway activation.

Meanwhile, chronic low-grade inflammation, another core characteristic of aging, was manifested in the significant upregulation of mRNA expression of pro-inflammatory cytokines such as IL-1β, TNF-α, and IL-6 in the liver and spleen of the model animals in this study [24]. Intervention with casein hydrolysate peptides dose-dependently reduced the expression of these inflammatory mediators. This observation is compatible with attenuation of ROS-sensitive inflammatory signaling, including NF-κB-associated pathways; however, NF-κB activation was not directly measured in the present study. In addition, an intestinal contribution cannot be excluded. Dietary bioactive peptides may interact with the gut environment and influence microbiota–immune communication, which could in turn affect systemic inflammatory status. Nevertheless, the intestinal microbiota and intestinal innate immune signaling were not examined here, and therefore a gut-mediated mechanism remains hypothetical. Future studies should characterize fecal microbiota using 16S rRNA gene sequencing or metagenomic approaches and assess intestinal Toll-like receptor signaling, particularly the TLR4/MyD88/NF-κB axis, by qPCR and/or protein-level analyses. Evaluation of intestinal barrier integrity and microbial metabolites would further help determine whether a gut–immune axis contributes to the effects of casein hydrolysate peptides [25,26]. It is worth noting that there may be a positive interaction between antioxidant and anti-inflammatory effects: after the hydrolyzed peptides alleviate oxidative stress, the continuous activation of NF-κB by reactive oxygen species is weakened, thereby inhibiting the inflammatory cascade at the transcriptional level and forming an “antioxidant-anti-inflammatory” virtuous cycle [27].

In terms of immune regulation, D-galactose-induced aging leads to a significant decrease in the immune organ index of the thymus and spleen in mice, an imbalance in the proportion of T lymphocyte subsets in the spleen, and abnormal fluctuations in serum immunoglobulin levels of IgM and IgG, reflecting the comprehensive manifestations of aging-related immune senescence [28]. After intervention with casein hydrolyzed peptides, the immune organ index was significantly restored, the proportion of CD4^+^/CD8^+^ T cell subsets tended to normalize, and the serum levels of IgM and IgG tended to shift toward those observed in the control group, suggesting a modulatory effect on aging-associated alterations in humoral immune parameters. However, these measurements do not provide direct evidence of immunoglobulin class switching, antigen-specific responses, or the establishment of humoral immune memory. Further studies assessing B-cell subsets, class-switch recombination, antigen-specific antibody responses, and memory B-cell formation are required to clarify these mechanisms [29]. This immunoregulatory effect may involve altered cytokine signaling and immune-cell homeostasis. A contribution from the gut microbiota–immune axis is also biologically plausible, but cannot be established from the present data because intestinal microbial composition and intestinal immune signaling were not assessed [30]. In addition, histopathological evaluation further confirmed the protective effect of casein hydrolyzed peptides on target organs: in aging model mice, hepatocytes around the central vein of the liver lobule showed significant swelling, degeneration, and inflammatory cell infiltration, while the granule cell layer of the hippocampal dentate gyrus became thinner, neuronal density decreased, and karyopyknosis occurred [31]; after intervention with hydrolyzed peptides, the structural damage of the aforementioned liver and brain tissues was alleviated in a dose-dependent manner, and the high-dose group approached the level of the normal control group [32]. As the core organ of metabolism and detoxification in the body, the alleviation of inflammation in the liver helps reduce systemic inflammatory burden; while the hippocampal dentate gyrus is a key brain region for neurogenesis and cognitive function, the protection of its structural integrity suggests that hydrolyzed peptides have potential application prospects in preventing age-related neurodegenerative diseases. This protective effect is mainly attributed to their synergistic promotion of tissue repair through antioxidant and anti-inflammatory activities, as well as improved immune function [33,34]. Another mechanistic issue concerns cellular senescence. Chronic D-galactose exposure has been reported to promote senescence-associated phenotypes through oxidative stress [35], DNA damage, and activation of cell-cycle arrest pathways such as p53/p21 and p16^INK4a^, accompanied in some models by an enhanced senescence-associated secretory phenotype (SASP) [36]. Because senescent cells can themselves contribute to persistent inflammatory signaling, cellular senescence may represent an important link between the oxidative and inflammatory abnormalities observed in the present model. However, the current study assessed histopathological injury and systemic/immune inflammatory parameters rather than cellular senescence directly. Thus, the improvements in liver and brain morphology and inflammatory status cannot be interpreted as direct evidence that casein hydrolysate peptides reduced the senescent-cell burden. Future studies should therefore quantify cellular senescence in brain, liver, and intestinal tissues using senescence-associated β-galactosidase staining together with immunohistochemical or immunofluorescence detection of markers such as p16^INK4a^ and p21^Cip1^, and should evaluate relevant SASP factors. Such analyses would help determine whether modulation of cellular senescence contributes to the protective effects observed here.

Although this study comprehensively reveals the anti-aging effects and multi-target action characteristics of casein hydrolysate peptides at an overall level, several limitations should be considered when interpreting the present findings. First, the casein hydrolysate used here is a complex peptide mixture, and the individual peptide sequence(s) principally responsible for the observed biological effects have not been established [37]. Activity-guided fractionation and subsequent structure–activity studies are therefore required. Second, although the present biochemical and transcriptional findings are consistent with modulation of oxidative stress and inflammatory signaling, direct evidence for key upstream pathways, including Nrf2-ARE, MAPK/NF-κB, AGE/RAGE, and apoptosis-related signaling, was not obtained [38]. Third, the possibility that D-galactose interacts directly with casein-derived peptides in vivo remains unresolved; quantitative determination of free D-galactose, circulating bioavailable peptides, peptide–D-galactose reaction products, and AGE formation will be necessary to evaluate this hypothesis. Fourth, the involvement of the gut microbiota and intestinal innate immune signaling remains to be established by microbiome profiling and analysis of intestinal TLR-related pathways [39,40]. Finally, although D-galactose is widely used to induce aging-like phenotypes, cellular senescence was not directly quantified in the present study. Future studies incorporating SA-β-gal analysis and tissue-specific detection of p16^INK4a^, p21^Cip1^, and SASP markers in the liver, brain, and intestine will be necessary to determine the contribution of senescent cells. These mechanistic studies, together with validation in naturally aged animals, will further clarify the biological basis and translational relevance of the effects observed here [41,42].

On the other hand, the choice of animal model should also be considered when interpreting the present findings. The D-galactose-induced model is an accelerated aging-like model rather than a model of physiological aging. Its advantages include a relatively short induction period, good experimental controllability, and the ability to reproduce several aging-associated features, including oxidative stress, chronic inflammation, immune dysfunction, and tissue injury. However, it cannot fully recapitulate the progressive, multifactorial changes that occur during natural aging. Naturally aged mice and senescence-accelerated strains such as SAMP8 may therefore provide complementary validation in future studies. In addition, only male mice were included in the present study. This design reduced variability associated with sex-specific reproductive endocrine changes but precluded assessment of sex-dependent responses to D-galactose and casein hydrolysate peptides. Future studies should include both sexes and naturally aged or complementary accelerated-aging models to evaluate the generalizability of the present findings.

## 5. Conclusions

Casein hydrolysate peptides exert significant anti-aging effects in D-galactose-induced aging mice, as evidenced by improved general physiological status, dose-dependent alleviation of systemic oxidative stress through enhanced antioxidant enzyme activities and reduced lipid peroxidation, and downregulation of pro-inflammatory cytokine expression. Histopathological analysis confirmed protection against hepatic and cerebral tissue damage. Concurrently, peptide treatment restored immune organ indices, corrected lymphocyte subset imbalances, and normalized serum immunoglobulin levels, indicating comprehensive immunoregulatory activity. These multi-target protective effects are likely mediated through coordinated regulation of oxidative stress, inflammation, and immune function, with possible involvement of the Nrf2-ARE pathway, gut microbiota, and telomere maintenance. Collectively, our findings establish casein-derived peptides as promising candidates for further mechanistic investigation and translational evaluation in aging biology.

## Figures and Tables

**Table 1:** Primer sequences used for quantitative real-time PCR analysis.

Gene	Forward Primer (5′–3′)	Reverse Primer (5′–3′)	Product Size (bp)
β-Actin	CACTGTCGAGTCGCGTCC	TCATCCATGGCGAACTGGTG	89
IL-6	AGAGGAGACTTCACAGAGGATAC	TCATTTCCACGATTTCCCAGAG	103
TNF-α	GCCAACGGCATGGATCTCAA	TCTTGACGGCAGAGAGGAGG	181

**Table 2:** Numbers and Percent of Quantitative Peptides in the Sample.

Detection Items	Numbers/Percent
Total number of peptides	1796
Number of phosphopeptides	250
Number of functional peptides	72
Percentage of phosphopeptides	13.9%
Percentage of functional peptides	4.0%
Relative intensity of phosphopeptides in the sample	11.4%
Relative intensity of other functional peptides in the sample	23.6%
Relative intensity of functional peptides in the sample	35.0%

Summary of the total number, functional classification, and relative intensity distribution of peptides detected via LC-MS in the analyzed sample. Percentages and relative intensities reflect the proportion of specific peptide subsets relative to the total identified peptides.

**Table 3:** Quantitative and Functional Characterization of Peptides Identified in the Sample.

The Names of Peptides	Relative MS Signal Intensity (%)	Molecular Weight	Potential Functions
YQEPVLGPVRGPFPIIV	10.17%	1880 Da	ACE-inhibitory/Anticancer/Antimicrobial/Antithrombotic/Immunomodulatory
TDVENLHLPLPL	4.27%	1360 Da	Not Found
GTQYTDAPSFSDIPNPIGSENSEKTTMPLW	3.9%	3283 Da	ACE-inhibitory
QEPVLGPVRGPFPIIV	3.6%	1717 Da	ACE-inhibitory
GVSKVKEAMAPK	2.5%	1244 Da	Not Found
LYQEPVLGPVRGPFPIIV	0.83%	1994 Da	Immunomodulatory

Relative MS signal intensity represents the normalized signal detected by LC-MS/MS and should not be interpreted as absolute peptide abundance, as MS response may vary depending on peptide sequence, ionization efficiency, and matrix effects.

## Data Availability

The data presented in this study are only available on request from the corresponding author due to the fact that they are part of an ongoing research project that has not yet been concluded, and the full dataset remains under active validation and analysis as part of the unfinished work; premature release of the data may affect the integrity of the project and related intellectual property outcomes.

## Supplementary Materials

The following supporting information can be downloaded at: https://www.mdpi.com/article/10.3390/biology15191741/s1, Table S1: Sensitivity analysis for comparisons based on three biological replicates per group; Table S2: Effect sizes and 95% confidence intervals for oxidative stress markers shown in Figure 4; Table S3: Effect sizes and 95% confidence intervals for inflammatory markers shown in Figure 5; Table S4: Effect sizes and 95% confidence intervals for serum immunoglobulin levels shown in Figure 11; Table S5: Effect sizes and 95% confidence intervals for hepatic inflammatory gene expression (Liver) shown in Figure 12; Table S6: Effect sizes and 95% confidence intervals for hepatic inflammatory gene expression (Spleen) shown in Figure 13.
