# Supplementary material for: Casein Hydrolysate Peptides Attenuate Immunosenescence and Inflammaging in a D-Galactose-Induced Mouse Model of Aging
Source: Biology (Basel). 2026 Oct 1;15(19):1741. doi: 10.3390/biology15191741 (PMC13663542; doi:10.3390/biology15191741)
Supplement: Supplementary file 1 [file biology-15-01741-s001.zip › biology-4483861-supplementary.pdf]

Casein Hydrolysate Peptides Attenuate Immunosenescence and Inflammaging in a D-Galactose-Induced Mouse Model of Aging

Menngi Cui, Zheng Xu, Jianbo Cheng, Jikun Zhang, Caihong Hu and Yun Zhang

Table S1. Sensitivity analysis for comparisons based on three biological replicates per group.

Parameter	Value
Biological replicates per group	3
Comparison framework	Two independent groups
Test direction	Two-sided
Significance level, α	0.05
Target statistical power	0.80
Minimum detectable standardized effect size	Cohen's d = 3.07

The minimum detectable effect size was estimated for a two-sided independent two-sample comparison with equal group sizes (n = 3 per group), α = 0.05, and 80% power. This sensitivity analysis characterizes the statistical resolution of analyses based on three biological replicates and should not be interpreted as a retrospective demonstration that all individual comparisons were adequately powered.

Table S2. Effect sizes and 95% confidence intervals for oxidative stress markers shown in Figure 4.

Tissue	Marker	Comparison	Mean difference (95% CI)	Hedges' g (95% CI)
Liver	SOD	BC vs AM	94.93 (84.73, 105.12)	25.83 (7.86, 43.81)
	SOD	PC vs AM	71.90 (63.35, 80.45)	23.22 (7.05, 39.38)
	SOD	LD vs AM	16.03 (10.51, 21.55)	7.84 (2.18, 13.51)
	SOD	MD vs AM	33.86 (29.71, 38.01)	21.48 (6.51, 36.45)
	SOD	HD vs AM	68.31 (67.43, 69.19)	143.25 (43.97, 242.53)
	GSH-Px	BC vs AM	444.79 (400.87, 488.72)	27.05 (8.24, 45.87)
	GSH-Px	PC vs AM	305.18 (246.52, 363.83)	14.19 (4.23, 24.15)
	GSH-Px	LD vs AM	84.04 (59.13, 108.95)	8.35 (2.35, 14.35)
	GSH-Px	MD vs AM	151.55 (136.55, 166.55)	22.21 (6.74, 37.68)
	GSH-Px	HD vs AM	301.57 (290.37, 312.77)	54.42 (16.68, 92.16)
	CAT	BC vs AM	34.60 (33.39, 35.81)	67.49 (20.70, 114.29)
	CAT	PC vs AM	22.56 (20.64, 24.47)	30.29 (9.24, 51.33)
	CAT	LD vs AM	4.82 (4.29, 5.34)	17.31 (5.21, 29.41)
	CAT	MD vs AM	9.52 (9.07, 9.96)	38.78 (11.86, 65.71)
	CAT	HD vs AM	21.62 (21.23, 22.01)	102.04 (31.31, 172.76)
	MDA	BC vs AM	−7.49 (−7.72, −7.25)	−80.05 (−135.55, −24.56)
	MDA	PC vs AM	−5.23 (−5.46, −4.99)	−55.38 (−93.79, −16.97)
	MDA	LD vs AM	−1.91 (−2.38, −1.44)	−8.84 (−15.16, −2.51)
	MDA	MD vs AM	−3.93 (−4.30, −3.57)	−21.55 (−36.56, −6.53)
	MDA	HD vs AM	−5.24 (−5.47, −5.01)	−41.12 (−69.66, −12.58)
Spleen	SOD	BC vs AM	103.07 (95.47, 110.68)	31.06 (9.48, 52.64)
	SOD	PC vs AM	83.92 (74.28, 93.56)	21.06 (6.38, 35.75)
	SOD	LD vs AM	30.41 (24.60, 36.21)	11.15 (3.26, 19.05)
	SOD	MD vs AM	57.70 (47.59, 67.82)	13.94 (4.15, 23.73)
	SOD	HD vs AM	81.10 (71.70, 90.50)	20.77 (6.29, 35.25)
	GSH-Px	BC vs AM	255.01 (247.72, 262.30)	67.96 (20.84, 115.08)

Tissue	Marker	Comparison	Mean difference (95% CI)	Hedges' g (95% CI)
	GSH-Px	PC vs AM	225.89 (198.23, 253.54)	20.05 (6.07, 34.04)
	GSH-Px	LD vs AM	42.12 (34.63, 49.61)	10.38 (3.01, 17.75)
	GSH-Px	MD vs AM	121.00 (112.73, 129.27)	26.55 (8.08, 45.02)
	GSH-Px	HD vs AM	221.67 (214.36, 228.98)	60.83 (18.65, 103.01)
	CAT	BC vs AM	22.97 (21.04, 24.90)	32.61 (9.96, 55.27)
	CAT	PC vs AM	17.62 (15.03, 20.21)	18.85 (5.69, 32.01)
	CAT	LD vs AM	7.58 (5.44, 9.71)	9.77 (2.81, 16.73)
	CAT	MD vs AM	10.97 (10.73, 11.20)	85.65 (26.28, 145.02)
	CAT	HD vs AM	17.54 (16.06, 19.03)	31.88 (9.73, 54.03)
	MDA	BC vs AM	−5.32 (−5.95, −4.70)	−18.02 (−30.60, −5.43)
	MDA	PC vs AM	−4.19 (−4.93, −3.45)	−15.76 (−26.79, −4.72)
	MDA	LD vs AM	−2.17 (−2.78, −1.55)	−6.70 (−11.60, −1.79)
	MDA	MD vs AM	−3.26 (−3.95, −2.58)	−11.85 (−20.22, −3.48)
	MDA	HD vs AM	−4.20 (−4.89, −3.51)	−15.34 (−26.09, −4.59)
Brain	SOD	BC vs AM	58.16 (52.61, 63.71)	28.73 (8.76, 48.71)
	SOD	PC vs AM	43.22 (37.94, 48.50)	22.40 (6.80, 38.01)
	SOD	LD vs AM	14.18 (7.26, 21.10)	5.66 (1.43, 9.90)
	SOD	MD vs AM	28.70 (23.96, 33.44)	16.46 (4.94, 27.98)
	SOD	HD vs AM	39.61 (32.70, 46.51)	15.86 (4.76, 26.97)
	GSH-Px	BC vs AM	267.00 (243.99, 290.01)	26.96 (8.21, 45.70)
	GSH-Px	PC vs AM	184.63 (162.83, 206.43)	15.82 (4.74, 26.90)
	GSH-Px	LD vs AM	76.34 (53.57, 99.12)	6.11 (1.58, 10.63)
	GSH-Px	MD vs AM	134.25 (111.79, 156.71)	13.29 (3.94, 22.64)
	GSH-Px	HD vs AM	184.01 (162.50, 205.52)	16.73 (5.03, 28.44)
	CAT	BC vs AM	17.22 (16.39, 18.05)	37.50 (11.47, 63.54)
	CAT	PC vs AM	12.33 (11.56, 13.09)	32.35 (9.87, 54.82)
	CAT	LD vs AM	5.70 (4.77, 6.64)	11.18 (3.27, 19.08)
	CAT	MD vs AM	8.54 (7.72, 9.36)	18.83 (5.69, 31.98)
	CAT	HD vs AM	11.93 (10.29, 13.57)	15.53 (4.65, 26.40)
	MDA	BC vs AM	−8.24 (−8.85, −7.63)	−29.07 (−49.27, −8.86)
	MDA	PC vs AM	−6.17 (−6.87, −5.48)	−16.18 (−27.51, −4.86)
	MDA	LD vs AM	−1.84 (−2.46, −1.23)	−5.50 (−9.64, −1.37)
	MDA	MD vs AM	−3.51 (−4.10, −2.91)	−11.59 (−19.77, −3.40)
	MDA	HD vs AM	−6.13 (−6.99, −5.26)	−13.49 (−22.98, −4.01)

Data are based on three biological replicates per group. Mean differences were calculated as the value in the indicated comparison group minus that in the AM group. Ninety-five percent confidence intervals for mean differences were calculated using Welch's method. Standardized effect sizes are presented as Hedges'g, which includes a small-sample correction. Approximate 95% confidence intervals are provided for Hedges'g. Because of the limited sample size and the very small within-group variability observed for several endpoints, some standardized effect-size estimates are unusually large and should therefore be interpreted cautiously.

Table S3. Effect sizes and 95% confidence intervals for inflammatory markers shown in Figure 5.

Marker	Comparison	Mean difference (95% CI)	Hedges' g (95% CI)
IL-6	BC vs. AM	−46.58 (−48.80, −44.36)	−43.80 (−74.20, −13.41)
IL-6	PC vs. AM	−39.01 (−40.36, −37.67)	−53.09 (−89.91, −16.26)
IL-6	LD vs. AM	−11.64 (−14.32, −8.95)	−9.55 (−16.35, −2.74)
IL-6	MD vs. AM	−25.37 (−27.03, −23.72)	−29.40 (−49.84, −8.96)
IL-6	HD vs. AM	−35.57 (−37.72, −33.42)	−34.28 (−58.10, −10.47)
IL-1β	BC vs. AM	−42.86 (−44.27, −41.45)	−55.87 (−94.62, −17.12)
IL-1β	PC vs. AM	−36.00 (−37.32, −34.67)	−49.52 (−83.87, −15.17)
IL-1β	LD vs. AM	−14.34 (−15.51, −13.16)	−26.93 (−45.65, −8.20)
IL-1β	MD vs. AM	−23.71 (−24.88, −22.54)	−37.23 (−63.08, −11.38)
IL-1β	HD vs. AM	−34.47 (−35.97, −32.96)	−42.74 (−72.41, −13.08)
TNF-α	BC vs. AM	−23.63 (−26.07, −21.20)	−23.09 (−39.17, −7.01)
TNF-α	PC vs. AM	−18.40 (−20.67, −16.12)	−16.86 (−28.64, −5.07)
TNF-α	LD vs. AM	−7.10 (−9.41, −4.79)	−5.65 (−9.87, −1.42)
TNF-α	MD vs. AM	−11.72 (−14.14, −9.30)	−11.40 (−19.45, −3.34)
TNF-α	HD vs. AM	−16.19 (−18.63, −13.74)	−15.86 (−26.97, −4.75)

Data are based on three biological replicates per group. Mean differences were calculated as the value in the indicated comparison group minus that in the AM group. Ninety-five percent confidence intervals for mean differences were calculated using Welch's method. Standardized effect sizes are presented as Hedges'g with a small-sample correction, together with approximate 95% confidence intervals. Negative values indicate lower inflammatory-marker levels relative to the AM group. Because of the limited sample size and the small within-group variability, some standardized effect-size estimates are very large and should be interpreted cautiously.

Table S4. Effect sizes and 95% confidence intervals for serum immunoglobulin levels shown in Figure 11.

Marker	Comparison	Mean difference (95% CI)	Hedges' g (95% CI)
IgG (ng/mL)	BC vs. AM	−0.7573 (−0.7820, −0.7326)	−75.41 (−127.68, −23.14)
	PC vs. AM	−0.7097 (−0.7339, −0.6855)	−69.76 (−118.12, −21.40)
	LD vs. AM	−0.2808 (−0.3091, −0.2525)	−18.26 (−30.98, −5.54)
	MD vs. AM	−0.4908 (−0.5149, −0.4667)	−48.11 (−81.48, −14.75)
	HD vs. AM	−0.6805 (−0.7047, −0.6563)	−66.89 (−113.26, −20.52)
IgM (pg/mL)	BC vs. AM	−35.1660 (−39.0621, −31.2699)	−20.02 (−33.95, −6.09)
	PC vs. AM	−25.6428 (−30.1049, −21.1807)	−15.74 (−26.71, −4.76)
	LD vs. AM	−9.5174 (−13.9026, −5.1322)	−5.80 (−10.02, −1.58)
	MD vs. AM	−14.4456 (−18.6703, −10.2209)	−8.66 (−14.80, −2.53)
	HD vs. AM	−24.0861 (−27.8282, −20.3440)	−12.86 (−21.86, −3.86)

Data are based on three biological replicates per group. Mean differences were calculated as the value in the indicated comparison group minus that in the AM group. Ninety-five percent confidence intervals for mean differences were calculated using Welch's method. Standardized effect sizes are presented as Hedges' g with a small-sample correction, together with approximate 95% confidence intervals. Negative values indicate lower serum immunoglobulin levels relative to the AM group. Because of the limited sample size and the small within-group variability, the standardized effect-size estimates are large and should be interpreted cautiously.

Table S5. Effect sizes and 95% confidence intervals for hepatic inflammatory gene expression (Liver) shown in Figure 12.

Marker	Comparison	Mean difference (95% CI)	Hedges' g (95% CI)
IL-6	BC vs. AM	−3.6695 (−3.7601, −3.5789)	−92.42 (−156.47, −28.36)
	PC vs. AM	−3.2138 (−3.3858, −3.0418)	−48.31 (−81.81, −14.81)
	LD vs. AM	−1.6221 (−1.6558, −1.5884)	−101.57 (−171.97, −31.18)
	MD vs. AM	−2.1058 (−2.2418, −1.9698)	−38.57 (−65.34, −11.81)
	HD vs. AM	−3.0570 (−3.1675, −2.9465)	−66.16 (−112.03, −20.30)
TNF-α	BC vs. AM	−4.5023 (−4.6206, −4.3840)	−69.15 (−117.09, −21.21)
	PC vs. AM	−3.7997 (−4.1022, −3.4972)	−28.67 (−48.57, −8.76)
	LD vs. AM	−1.1837 (−1.2940, −1.0734)	−21.00 (−35.62, −6.39)
	MD vs. AM	−2.4661 (−2.5886, −2.3436)	−36.50 (−61.83, −11.17)
	HD vs. AM	−3.7800 (−3.9502, −3.6098)	−43.08 (−72.96, −13.20)

Data are based on three biological replicates per group. Mean differences were calculated as the value in the indicated comparison group minus that in the AM group. Ninety-five percent confidence intervals for mean differences were calculated using Welch's method. Standardized effect sizes are presented as Hedges'g with a small-sample correction, together with approximate 95% confidence intervals. Negative values indicate lower relative gene-expression levels than those in the AM group. Because of the small number of biological replicates and the very low within-group variability for several measurements, standardized effect-size estimates are unusually large and should be interpreted cautiously.

Table S6. Effect sizes and 95% confidence intervals for hepatic inflammatory gene expression (Spleen) shown in Figure 13.

Marker	Comparison	Mean difference (95% CI)	Hedges' g (95% CI)
IL-6	BC vs. AM	−3.4122 (−3.4985, −3.3259)	−72.01 (−121.94, −22.09)
	PC vs. AM	−2.9774 (−3.1075, −2.8473)	−46.27 (−78.37, −14.17)
	LD vs. AM	−1.7098 (−1.7835, −1.6361)	−47.51 (−80.47, −14.55)
	MD vs. AM	−2.5296 (−2.6380, −2.4212)	−44.75 (−75.80, −13.70)
	HD vs. AM	−2.8582 (−3.0155, −2.7009)	−38.83 (−65.78, −11.87)
TNF-α	BC vs. AM	−3.1826 (−3.2578, −3.1074)	−81.34 (−137.74, −24.95)
	PC vs. AM	−2.9816 (−3.0594, −2.9038)	−83.81 (−141.91, −25.71)
	LD vs. AM	−1.1590 (−1.2373, −1.0807)	−32.77 (−55.54, −10.01)
	MD vs. AM	−1.9934 (−2.0763, −1.9105)	−43.63 (−73.91, −13.35)
	HD vs. AM	−2.8608 (−2.9360, −2.7856)	−74.31 (−125.83, −22.79)

Data are based on three biological replicates per group. Mean differences were calculated as the value in the indicated comparison group minus that in the AM group. Ninety-five percent confidence intervals for mean differences were calculated using Welch's method. Standardized effect sizes are presented as Hedges'g with a small-sample correction, together with approximate 95% confidence intervals. Negative values indicate lower relative mRNA expression than that in the AM group. Because of the limited sample size and the very low within-group variability, standardized effect-size estimates are unusually large and should be interpreted cautiously.
